# Comparative genomic analysis of antibiotic resistance and virulence genes in *Staphylococcus aureus* isolates from patients and retail meat

**DOI:** 10.3389/fcimb.2023.1339339

**Published:** 2024-01-12

**Authors:** Dalal M. Alkuraythi, Manal M. Alkhulaifi, Abdulwahab Z. Binjomah, Mohammed Alarwi, Mohammed I. Mujallad, Saleh Ali Alharbi, Mohammad Alshomrani, Takashi Gojobori, Sulaiman M. Alajel

**Affiliations:** ^1^Department of Botany and Microbiology, College of Science, King Saud University, Riyadh, Saudi Arabia; ^2^Department of Biology, College of Science, University of Jeddah, Jeddah, Saudi Arabia; ^3^Microbiology Department, Riyadh Regional Laboratory, Ministry of Health, Riyadh, Saudi Arabia; ^4^College of Medicine, Al-Faisal University, Riyadh, Saudi Arabia; ^5^Computational Bioscience Research Center, Biological and Environmental Sciences and Engineering, King Abdullah University of Science and Technology, Thuwal, Saudi Arabia; ^6^Department of Microbiology, Food and Drug Authority, Jeddah, Saudi Arabia; ^7^Reference Laboratory for Microbiology, Executive Department for Reference Laboratories, Research and Laboratories Sector, Food and Drug Authority, Riyadh, Saudi Arabia

**Keywords:** *Staphylococcus aureus*, MRSA, antibiotic resistance genes, virulence factors, Staphylococcal toxins

## Abstract

**Introduction:**

*Staphylococcus aureus* is a significant human pathogen that poses a threat to public health due to its association with foodborne contamination and a variety of infections. The factors contributing to the pathogenicity of *S. aureus* include virulence, drug resistance, and toxin production, making it essential to monitor their prevalence and genetic profiles. This study investigated and compared the genomic characteristics of *S. aureus* isolates from retail meat and patients in Saudi Arabia.

**Methods:**

A total of 136 *S. aureus* isolates were obtained between October 2021 and June 2022:84 from patients and 53 from meat samples in Riyadh, Saudi Arabia. *S. aureus* isolates were identified using conventional methods and MALDI-TOF MS, and methicillin-resistant *S. aureus* (MRSA) was identified using VITEK2 and BD Phoenix systems. MRSA was confirmed phenotypically using chromogenic agar, and genotypically by detecting *mec*A. Genomic data were analyzed using BactopiaV2 pipeline, local BLAST, and MLST databases.

**Results:**

Antibiotic resistance genes were prevalent in both meat and patient *S. aureus* isolates, with high prevalence of *tet*38, *bla*Z, and *fos*B. Notably, all *S. aureus* isolates from patients carried multidrug-resistant (MDR) genes, and a high percentage of *S. aureus* isolates from meat also harbored MDR genes. Phenotypically, 43% of the *S. aureus* isolates from meat and 100% of the patients’ isolates were MDR. Enterotoxin genes, including *sel*X, *sem*, and *sei*, exhibited high compatibility between meat and patient *S. aureus* isolates. Virulence genes such as *cap, hly/hla, sbi*, and *isd* were found in all *S. aureus* isolates from both sources.

**Conclusion:**

Our study established a genetic connection between *S. aureus* isolates from meat and patients, showing shared antibiotic resistance and virulence genes. The presence of these genes in meat derived isolates underscores its role as a reservoir. Genomic relatedness also suggests potential transmission of resistance between different settings. These findings emphasize the necessity for a comprehensive approach to monitor and control *S. aureus* infections in both animals and humans.

## 1 Introduction

*Staphylococcus aureus* is an opportunistic bacterium found in humans and animals, especially food-producing animals (Kadariya et al., 2014). These bacteria can enter the food chain during collection, slaughter, processing, packaging, and storage (Wu et al., 2018). It has been detected in processed cheese, raw beef, camel meat, and dairy products in Saudi Arabia (El Sheikha, 2016). Moreover, it is a major pathogen that causes a wide range of infections in community and hospital settings (Turner et al., 2019). *S. aureus* has a variety of virulence factors that significantly influence its clinical manifestations (Jenul and Horswill, 2019). For instance, surface adhesins, including clumping factors and fibronectin-binding proteins, promote tissue adherence and contribute to skin and soft tissue infections (Algammal et al., 2020). Other virulence factors, including toxins such as hemolysins, leukocidins [e.g., panton-valentine leukocidin (PVL)], enterotoxins, and exfoliative toxins, lead to diverse clinical outcomes, ranging from severe skin infections to food poisoning and scalded skin syndrome (Algammal et al., 2020). Staphylococcal protein A (SPA) interferes with the host immune response and increases the severity of staphylococcal infection (Algammal et al., 2020). Proteins that promote biofilm production, such as polysaccharide intercellular adhesins, contribute to chronic infections and antibiotic resistance, particularly in cases involving medical devices (Singh et al., 2009). These virulence factors contribute to *S. aureus* pathogenicity (Jenul and Horswill, 2019). Enterotoxigenic *S. aureus* strains produce heat-stable staphylococcal enterotoxins (SEs), which are one of the main causes of global outbreaks of food poisoning (Pinchuk et al., 2010). To date, more than 20 SEs have been identified (Sankomkai et al., 2020). Classically known enterotoxins include the five enterotoxins encoded by *sea*, *seb*, *sec*, *sed*, and *see* (Sankomkai et al., 2020). Beyond food poisoning, these proteins can contribute to a broad range of symptoms ranging from mild toxin-mediated illnesses to life-threatening systemic diseases (Ahmad-Mansour et al., 2021). According to a recent report by the European Food Safety Authority, more than 40 staphylococcal food poisoning (SFP) outbreaks, 400 cases, and 32 hospitalizations have been linked to *S. aureus. aureus* in the European Union by 2020 (The European Union One Health 2020 Zoonoses Report, 2021). SFP accounts for approximately 241,000 hospitalizations annually due to illness in the United States (Scallan et al., 2018). As SFP are becoming more prevalent, food safety is increasingly considered a serious public health issue that affects all other safety aspects (Kadariya et al., 2014). The extensive use of antibiotics has led to the emergence of multidrug-resistant (MDR) bacteria, including genetically distinguished methicillin-resistant *S. aureus* (MRSA) strains associated with communities and animals, such as community-associated MRSA (CA-MRSA) and livestock-associated MRSA (LA-MRSA) (Fetsch et al., 2021). *S. aureus* strains exhibit antibiotic resistance due to specific genes that render them resistant to various antimicrobials, such as penicillins, tetracyclines, cephalosporins, aminoglycosides, and fluoroquinolones (Urban-Chmiel et al., 2022). The global increase in MDR is recognized as a public health threat (Algammal et al., 2022a). Recent studies have highlighted the emergence of MDR bacterial pathogens from various sources, emphasizing the importance of routine antimicrobial susceptibility testing to identify effective antibiotics and screen for emerging MDR strains (Algammal et al., 2020; Elmoslemany et al., 2021; Gordon et al., 2021; Algammal et al., 2022b). Despite sporadic reports of *S. aureus* toxins and drug resistance genes in Saudi retail foods, information on the prevalence, toxigenicity, and antibiotic resistance of *S. aureus* remains limited (Raji et al., 2016; Alghizzi and Shami, 2021; Alghizzi et al., 2021). Molecular genomic methods are essential tools for investigating the virulence factors, antimicrobial resistance, and toxin genes in *S. aureus. aureus*. In addition, it aids in elaborating the toxigenic and drug resistance potential of this microorganism in retail meats and hospitals. Meat and meat products have been found to be important *S. aureus* reservoirs and have been linked to various foodborne bacterial outbreaks (van Loo et al., 2007; Boost et al., 2013; Ge et al., 2017; Wu et al., 2018; Thwala et al., 2021). In Saudi Arabia, data on *S. aureus. aureus* in retail meat from different regions was limited (Abulreesh and Organji, 2011; Kay et al., 2013; El Sheikha, 2016; Raji et al., 2016; Shawish and Al-Humam, 2016; El-Ghareeb et al., 2019). Therefore, this study aimed to investigate and compare the genomic relatedness and distribution of resistance and virulence genes among clinical and meat *S. aureus* isolates. The genomic investigation of *S. aureus* isolates from meat and their relationship with clinical *S. aureus* isolates may be an initial step in establishing an effective strategy in Saudi Arabia for monitoring the emergence and spread of *S. aureus*.

## 2 Material and methods

### 2.1 Sample collection

From October 2021 to June 2022, 84 non-duplicate isolates of *S. aureus* were obtained from inpatients at the bacteriology department of the Riyadh Regional Laboratory and Blood Bank. A wide range of specimens, including urine midstream, tissue culture, wound swab, nasal swab, pus swab, groin swab, axilla swab, burn swab, eye swab, bronchial wash, sputum, ascites (peritoneal) fluid, tracheal aspirate, and blood culture samples, were collected for analysis in this study. For additional details on the specimens, please refer to **Supplementary Table 4 in Supplementary Material**. Additionally, 250 raw meat samples, including camels, beef, chicken, fish, and lamb, were collected from various meat retailers in Riyadh, Saudi Arabia. All meat samples were transported in sealed plastic wrap or original packaging in insulated coolers maintained at a stable temperature of 4°C with adequate ice packs to sustain a stable temperature throughout the transportation process to the Reference Laboratory of Microbiology at the Saudi Food and Drug Authority. Microbiological analysis was conducted within 24 h of sampling.

### 2.2 Ethical approval

Ethical approval (approval number H-01-R-053) for the study was obtained from the Institutional Review Board committee of King Saud Medical City, Riyadh, Saudi Arabia and the Institutional Biosafety and Bioethics Committee of King Abdullah University of Science and Technology (approval number 22IBEC051).

### 2.3 *Staphylococcus aureus* isolation and identification

Meat samples were homogenized in a stomacher using a sterile plastic bag. This involved 25 g of each meat sample and 225 mL of buffered peptone water with 6.5% NaCl. Petrifilm™ Staph Express count plate (3M™, St. Paul, MN, USA), a modified chromogenic Baird-Parker medium, was used as a selective and differential medium for *S. aureus* detection. The Petrifilm™ Staph Express disk contained toluidine blue O and DNA. DNase-positive organisms degrade DNA that reacts with toluidine blue O to form pink zones. This allowed for a distinction between *S. aureus* colonies and other staphylococci. Single *S. aureus* colonies were transferred to mannitol salt agar (Neogen; Lansing, MI, USA). All *S. aureus* isolates were identified and confirmed using the classical method, which included Gram staining, catalase testing, and coagulase testing (PROLEX™, Neston, UK). All *S. aureus* isolates were further identified by matrix-assisted laser desorption/ionization time-of-flight mass spectrometry (MALDI-TOF) (Bruker, Bremen, Germany) using the 70% formic acid protein extraction method.

### 2.4 Phenotypic and genotypic detection of methicillin-resistant *Staphylococcus aureus*

The identification of MRSA strains was carried out using VITEK2 (bioMérieux, Craponne, France) and BD Phoenix (BD Diagnostics, Franklin Lakes, NJ, USA). The instruments’ systems classify any *S. aureus* isolate as MRSA based on both oxacillin and cefoxitin minimum inhibitory concentrations (MICs) breakpoints, interpreted according to Laboratory Standards Institute (CLSI). For oxacillin, isolates were considered resistant with MICs ≥4 μg/ml, whereas for cefoxitin, isolates were considered resistant with MICs ≥8 μg/ml. Individual colonies of the *S. aureus* isolates were transferred to Harlequin MRSA chromogenic agar for the selective and differential detection of MRSA (Neogen, Lansing, MI, USA). This medium allowed MRSA to grow because it includes a cefoxitin (Fox) supplement. The α-glucosidase enzyme produced by *S. aureus* cleaves the chromogenic substrate, resulting in blue colonies.

DNA was extracted from the bacterial culture using the QIAGEN DNeasy Blood and Tissue Kit (Qiagen, Manchester, UK), following the manufacturer’s instructions. DNA purity was checked using a QIAxpert spectrophotometer (Qiagen, Manchester, UK), and DNA concentration was determined using a Qubit™ Flex Fluorometer device (Thermo Fisher Scientific, Waltham, MA, USA).

The presence of methicillin resistance was determined by detecting the *mec*A gene, which encodes for penicillin’s binding protein 2a (PBP2a). This was performed using a forward primer (5′-GTAGAAATGACTGAACGTCCGATAA-3′) and a reverse primer (5′-CCAATTCCACATTGTTTCCGTCTAA-3′), which amplified a 310 bp fragment (Parvin et al., 2021). The reaction was performed in 25 µL using DreamTaq™ 2X Green PCR Master Mix (Thermo Scientific, Waltham, MA, USA). The reaction setup included 1 µL of each (10 pmol) primer and 2 µL of DNA template (20 ng/µL). *S. aureus* (ATCC43300) was used as a positive control (ATCC, Manassas, Virginia, USA). The amplification process involved an initial denaturation step at 95°C for 4 min, followed by 30 cycles at 95°C for 45 s, 56°C for 1 min, and 72°C for 1 min. This was followed by a final extension step at 72°C for 4 min. The final PCR product was analyzed on a 1.6% agarose gel stained with ethidium bromide and run for 60 min at 80 volts in 1X tris borate buffer (TBE) (BIOBasic, Markham, ON, Canada). Gel visualization was performed using the Image Lab™ 6.1 Software (Bio-Rad, Hercules, CA, USA).

### 2.5 Antimicrobial susceptibility testing

The antimicrobial susceptibility testing (AST) of *S. aureus* isolates was evaluated using both the VITEK 2 System and BD Phoenix System. Testing followed manufacturers’ instructions and adhered to CLSI guidelines (Wayne, 2018). Shared antimicrobial agents between the two systems encompassed 15 classes, including *β*-lactam: cefoxitin (FOX) and oxacillin (OXA); aminoglycosides: gentamicin (GEN); fluoroquinolones: levofloxacin (LEV) and moxifloxacin (MOX); macrolide: erythromycin (ERY); lincosamides: clindamycin (CLI); oxazolidinone: linezolid (LIN); glycopeptide: teicoplanin (TEI) and vancomycin (VAN); tetracycline: tetracycline (TET); glycycline: tigecycline (TIG); phosphonic acids: fosfomycin (FOS); nitroheterocyclics: nitrofurantoin (NIT); fusidanes: fusidic acid (FUS); monoxycarbolic acids: mupirocin (MUP); ansamycin: rifampin (RIF); and folate pathway antagonists: sulphamethoxazole/trimethoprim (SXT). However, the BD Phoenix System lacked benzylpenicillin (PEN) and tobramycin (TOB) present in the VITEK 2 system. Additionally, the BD Phoenix System included more antimicrobial agents such as *β*-lactam: ampicillin (AMP), cefotaxime (CTX), ceftaroline (CPT), and penicillin G (PEN); fluoroquinolones: ciprofloxacin (CIP); and lipopeptides: daptomycin (DAP). The reference strain (ATCC33400) was used, and the results, including the MICs, were interpreted following CLSI guidelines and MIC breakpoints for *S. aureus (* Clinical and Laboratory Standards Institute, 2023). *S. aureus* isolates resistant to three or more antimicrobial classes or to oxacillin/cefoxitin were classified as MDR (Magiorakos et al., 2012).

### 2.6 Whole-genome sequencing

Whole-genome sequencings (WGS) libraries were constructed using a QIAseq DNA FX library preparation kit (Qiagen, Manchester, UK). The input concentration was 100 ng of DNA, following the manufacturer’s instructions (Qiagen, Manchester, UK). The library was size-selected to have a 300–350 bp insert size. Sequencing was performed using the Illumina NovaSeq 6000 platform with two SP flow cells (Illumina, San Diego, CA, USA). Data were quality-checked prior to analysis, with a Phred score cut-off of Q30. Bioinformatics analysis was conducted using the BactopiaV2 pipeline, focusing on the *S. aureus*-specific workflow (Petit and Read, 2020). Screening for *pvl* was carried out using local BLAST database identification of the two genes, *luk*S and *luk*F, accessions (YP_002268030.1 and YP_002268029.1), respectively. For sequence type (ST) assignment, the public MLST database for *S. aureus* was used. The clonal lineages of all *S. aureus* strains isolated from the patients and meat samples were identified using MLST.

### 2.7 Statistical analysis

To investigate the association between antibiotic resistance, toxins, and virulence genes in *S. aureus* isolates obtained from meat and patients, data were analyzed using GraphPad Prism 10.0 software (GraphPad Software, Boston, Massachusetts, USA). The two-tailed chi-square test (χ2) was used to determine the significance of the differences, and *p* values < 0.05 were considered statistically significant. The correlation coefficient (r) was calculated among the demonstrated antimicrobial resistance genes in the *S. aureus* strains and different antimicrobial agents. The Pearson correlation coefficient in a correlation matrix was estimated using the “corrplot” package in R, (version 4.2.1) (Wei and Simko, 2021).

## 3 Results

### 3.1 Prevalence of *Staphylococcus aureus* isolates

In the analysis of 250 meat samples, 53 isolates of *S. aureus* were detected, comprising 21.2% of the total meat samples (53/250). Further analysis revealed 136 *S. aureus* isolates, of which 83 (61%) were obtained from patients and 53 (39%) from meat samples. The breakdown of *S. aureus* isolates across the various meat types was as follows: camel meat (18/53; 34%), beef (12/53; 23%), lamb (8/53; 15%), fish (8/53; 15%), and chicken (7/53; 13%).

### 3.2 Phenotypic characteristics of the *Staphylococcus aureus* isolates

All *S. aureus* isolates recovered in this study were positive for several key phenotypic tests, such as Gram staining and catalase and coagulase tests. The isolates showed beta-hemolysis on blood agar, indicating the production of hemolysins and fermented mannitol on mannitol salt agar, leading to a color change from pink to yellow. Antibiotic susceptibility testing provided specific profiles for the sensitivity and resistance to various antibiotics. Using VITEK2, 56 *S. aureus* isolates from patients and 11 from meat were identified as MRSA, exhibiting positivity for cefoxitin and an oxacillin MIC ≥4 mg/L. The 42 methicillin-sensitive *S. aureus* (MSSA) isolates from meat were cefoxitin screen-negative, with oxacillin MICs measuring 0.5 mg/L. Additionally, BD Phoenix™ identified 26 *S. aureus* isolates from patients as MRSA, all demonstrating cefoxitin MIC ≥8 mg/L and oxacillin MIC >2 mg/L.

### 3.3 Phenotypic and genotypic resistance characteristics of *S. aureus* isolates

**Figure 1:**
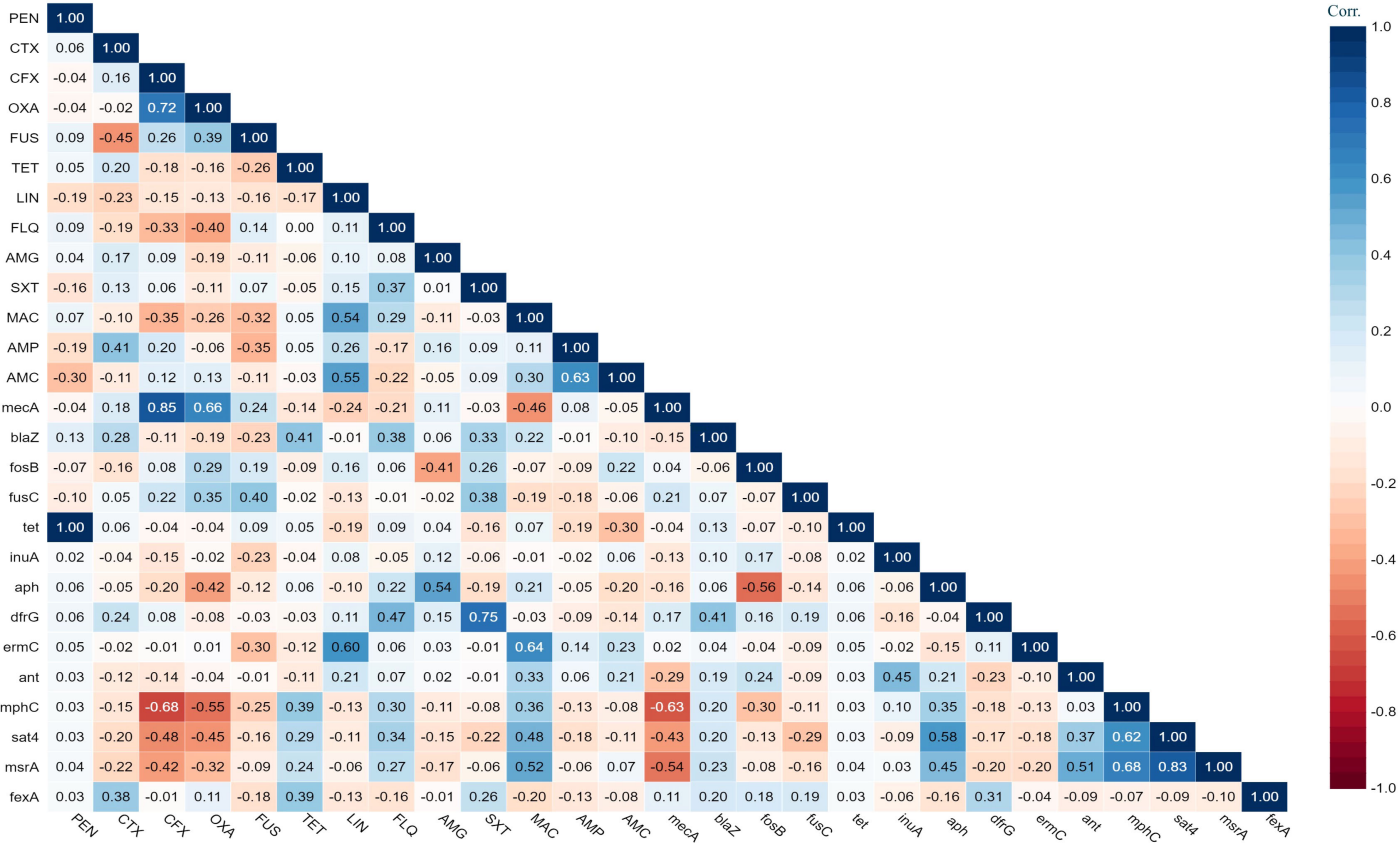
The heat map illustrates the correlation coefficient (r) between the demonstrated antimicrobial resistance genes in the *S. aureus* strains and different antimicrobial agents.

The antimicrobial susceptibility of the pooled *S. aureus* isolates were resistance to penicillin (97.7%), cefoxitin (68,3%), oxacillin (64,6%), levofloxacin and moxifloxacin (33.8%), erythromycin (27.9%), and trimethoprim/sulfamethoxazole (22%). Statistical analysis indicated no significant variation in susceptibility patterns among the *S. aureus* strains to different tested antimicrobial classes (p = 0.09). Among the meat isolates, 20.7% (11/53) demonstrated sensitivity to all antibiotics, while 35.8% (19/53) exhibited resistance (R) to penicillin (PEN) in the presence of *blaZ, fosB-Saur, and tet38* genes, coupled with a MAR index of 0.05. Furthermore, 43% (23/53) of the meat isolates were categorized as MDR, displaying resistance to at least one antimicrobial agent in a minimum of three categories, according to Magiorakos et al.’s criteria (Magiorakos et al., 2012). These MDR strains harbored *aph(3’)-IIIa, blaZ, mphC, msrA, sat4*,and *tet38* genes, and had a MAR index ranging from 0.15 to 0.3. Clinical *S. aureus* isolates exhibited diverse resistance patterns, with 100% demonstrating MDR and MAR indices ranging from 0.15 to 0.8, as shown in **Table 1**. The study revealed positive correlations between: *tet* gene with PEN (r=1); *mec*A with CFX (r=0.85); and *dfr*G with SXT (r=0.75), as depicted in **Figure 1**.

**Table 1:** The Prevalence of Multidrug-Resistance Profiles and the Resistance Genes Among the isolated S. aureus (n=136).

Source of *S. aureus* isolates	No. of strains %	Type of resistance S, R, MDR	Phenotypic multidrug resistance (classes and antibiotics)	The antibiotic-resistance genes	MAR index
Meat	3 (2.2)	R	PEN	*blaZ, tet38, tetK*	0.05
	8 (5.8)	S	_	*fosB-Saur, tet38*	_
	2 (1.4)	S	_	*tet38*	_
	1 (0.7)	S	_	*tet38, tetK*	_
	7 (5.1)	R	PEN	*blaZ, fosB-Saur, tet38*	0.05
	8 (5.8)	R	PEN	*blaZ, tet38*	0.05
	3 (2.2)	MDR	Two classes: CFX, PEN, OXA, and FUS	*mecA, aph(2’’)-Ih, fusC, tet38*	0.2
	1 (0.7)	R	Two classes: PEN, OXA, and FUS	*fosB-Saur, tet38*	0.15
	2 (1.4)	MDR	Five classes: PEN, CIP, ERY, CLI, and STX	*dfrG, ermC, fosB-Saur, tet38*	0.25
	1 (0.7)	MDR	Four Classes: PEN, CIP, GEN, and CLI	*ant(4’)-Ia, blaZ, fosB-Saur, lnuA, tet38, tetK*	0.2
	1 (0.7)	MDR	Three classes: PEN, ERY, and CLI	*blaZ, ermC, fosB-Saur, tet38*	0.15
	1 (0.7)	MDR	Four classes: PEN, CIP, ERY, CLI,	*ant (6*)*-Ia, aph(3’)-IIIa, blaZ, fosB-Saur, sat4, tet38*	0.2
	5 (3.6)	MDR	Four classes: PEN, CIP, ERY, and TET	*aph(3’)-IIIa, blaZ, mphC, msrA, sat4, tet38*	0.2
	1 (0.7)	MDR	Four classes: PEN, CIP, ERY, and TET	*aph(3’)-IIIa, blaZ, fusC, mphC, msrA, sat4, tet38*	0.2
	3 (2.2)	MDR	Four classes: CFX, PEN, OXA, CIP, TET, and FUS	*mecA, blaZ, fosB-Saur, tet38*	0.3
	1 (0.7)	MDR	Four classes: CFX, PEN, OXA, CIP, ERY, and STX	*mecA, blaZ, dfrG, ermC,fosB-Saur, fusC, tet38*	0.3
	1 (0.7)	MDR	Four classes: PEN, OXA, CIP, ERY, and STX	*ant(4’)-Ia,blaZ, fosB-Saur,fusB, lnuA, mphC, msrA,tet38*	0.25
	2 (1.4)	MDR	Three classes: CFX, PEN, OXA, TET, and FUS	*mecA, blaZ, fosB-Saur, fusC, tet38*	0.25
	1 (0.7)	MDR	Three classes: CFX, PEN, OXA, GEN, TET	*mecA, blaZ,erm (B), fexA,fosB-Saur, tet38, tetK, tetM*	0.2
	1 (0.7)	MDR	Two classes: CFX, PEN, OXA, and CLI	*mecA, blaZ, tet38,vga(A)-LC*	0.2
Clinical	1 (0.7)	MDR	Two classes: AMC, AMP, CFX, OXA, and PEN	*mecA, blaZ, fosB-Saur, tet38*	0.3
	1 (0.7)	MDR	Two classes: CFX, PEN, OXA, and FUS	*mecA, blaZ, fusC, tet38*	0.2
	4 (2.9)	MDR	Three classes: CFX, PEN, OXA, FUS, and CIP	*mecA, fosB-Saur, fusC, tet38*	0.25
	1 (0.7)	MDR	One class: CFX, PEN, and OXA	*mecA, fosB-Saur, fusC, tet38*	0.15
	5 (3.6)	MDR	Four classes: CTX, CFX, OXA, PEN, TET, and STX	*mecA, blaZ, dfrC, fexA, fosB-Saur, fusC, tet38, tetM*	0.3
	5 (3.6)	MDR	Three classes: CFX, PEN, OXA, FUS, and GEN	*mecA, aph(2’’)-Ih, fusC, tet38*	0.25
	10 (7.3)	MDR	Four classes: CFX, PEN, OXA, CIP, FUS, and STX	*mecA, blaZ, dfrG, fosB-Saur, fusC, tet38*	0.3
	1 (0.7)	MDR	10 classes: AMC, AMP, CIP, CFX, PEN, OXA, FUS, CLI, ERY, GEN, TET, RIF, TEI and SXT	*mecA, blaZ, dfrG, fosB-Saur, fusC, tet38*	0.8
	1 (0.7)	MDR	Two classes: CFX, PEN, OXA, and ERY	*mecA, ant(6)-Ia, aph(3’)-IIIa, fosB-Saur, msrA, sat4, tet38*	0.2
	1 (0.7)	MDR	Two classes: CFX, PEN, OXA, and FUS	*mecA, blaZ, fusB, tet38*	0.2
	1 (0.7)	MDR	Three classes: CFX, CTX, PEN, OXA, and GEN	*mecA, ant(4’)-Ia, aph(2’’)-Ih, blaZ, fosB-Saur,fusC, lnuA, tet38*	0.25
	2 (1.4)	MDR	Two classes: CFX, PEN, OXA, and FUS	*mecA, fusC, tet38*	0.2
	8 (5.8)	MDR	Two classes: CFX, PEN, OXA, and FUS	*mecA, fosB-Saur, tet38*	0.2
	2 (1.4)	MDR	Two classes: AMP, CTX, CFX, OXA, and PEN	*mecA, fosB-Saur, tet38*	0.25
	1 (0.7)	MDR	Three classes: AMP, CTX, CFX, OXA, PEN, and TET	*mecA, blaZ, fosB-Saur, tet38, tetK*	0.3
	1 (0.7)	MDR	Four classes: AMP, CTX, CFX, GEN, OXA, PEN, and TET	*mecA, aph(2’’)-Ih, aph(3’)-IIIa, blaZ, fusC, tet38, tetL*	0.3
	1 (0.7)	MDR	Five classes: AMC, AMP, CFX, CLI, ERY, OXA, PEN, and TET	*mecA, ant(4’)-Ia, blaZ, ermC, fosB-Saur, lnuA, tet38, tetK*	0.45
	4 (2.9)	MDR	Four classes: AMC, AMP, CFX, CLI, ERY, OXA, and PEN	*mecA, ermC, fosB-Saur, tet38*	0.4
	1 (0.7)	MDR	Three classes: AMP, CTX, CFX, OXA, PEN, and TET	*mecA, blaZ, fusC, tet38, tetK*	0.3
	1 (0.7)	MDR	Four classes: AMP, CTX, CFX, ERY, OXA, PEN, and TET	*mecA, blaZ, ermC fusC, tet38, tetK*	0.35
	1 (0.7)	MDR	Four classes: CFX, PEN, OXA, CIP, ERY, and TET	*mecA, aph(3’)-IIIa, blaZ, dfrG, fosB-Saur, mphC, msrA, sat4, tet38, tetK*	0.3
	4 (2.9)	MDR	Five classes: AMP, CTX, CFX, GEN, CIP, PEN, and STX	*mecA, aph(2’’)-Ih, blaZ, dfrC, tet38*	0.4
	1 (0.7)	MDR	Two classes: AMP, CTX, CFX, OXA, and PEN	*mecA, tet38*	0.25
	3 (2.2)	MDR	Four classes: CFX, PEN, OXA, CIP, ERY, and FUS	*mecA, aph(3’)-IIIa, blaZ, fosB-Saur, mphC, msrA, sat4, tet38*	0.3
	1 (0.7)	MDR	Five classes: CFX, PEN, OXA, CIP, ERY, CLI, and FUS	*mecA, ermC, fosB-Saur, fusC, tet38*	0.35
	3 (2.2)	MDR	Five classes: CFX, PEN, OXA, GEN, CIP, ERY, and CLI	*mecA, aph(2’’)-Ih, blaZ, dfrC, ermC, tet38*	0.35
	1 (0.7)	MDR	Three classes: CTX, PEN, OXA, CIP, and FUS	*mecA, blaZ, dfrG, fexA, fosB-Saur, fusC, tet38, tetL*	0.25
	1 (0.7)	MDR	Six classes: AMC, AMP, CFX, CLI, LZD, MUP, OXA, PEN, and STX	*mecA, fosB-Saur, fusC, tet38*	0.5
	1 (0.7)	MDR	Four classes: AMP, CTX, CFX, CIP, ERY, OXA, and PEN	*mecA, blaZ, ermC, tet38*	0.35
	2 (1.4)	MDR	Five classes: AMC, AMP, ERY, CLI, CFX, FUS, OXA, PEN, and STX	*ant(4’)-Ia, blaZ, bleO, fosB-Saur, fusB, msrA, tet38*	0.35
	2 (1.4)	MDR	Two classes:CTX, CFX, OXA, and PEN	*mecA, blaZ, dfrG, fosB-Saur, tet38*	0.2
	3 (2.2)	MDR	Three classes:CTX, CFX, ERY, OXA, and PEN	*mecA, blaZ, ermC, fusC, tet38*	0.25
	1 (0.7)	MDR	One class: CFX, PEN, and OXA	*mecA, fosB-Saur, tet38*	0.15
	1 (0.7)	MDR	One class: CFX, PEN, and OXA	*mecA, fosB-Saur, lnuA, tet38*	0.15
	3 (2.2)	MDR	Six classes: CFX, PEN, OXA, CIP, ERY, CLI, FUS, and STX	*mecA, blaZ, dfrG, ermC, fosB-Saur, fusC, tet38*	0.4
	1 (0.7)	MDR	One class: CFX, PEN, and OXA	*mecA, blaZ, fosB-Saur, lnuA, tet38*	0.15
	1 (0.7)	MDR	Two classes: CFX, PEN, OXA, and TET	*mecA, blaZ, tet38, tetK*	0.2

Classes and antibiotics: *β*-lactams and cephalosporins: benzylpenicillins (PEN), oxacillin (OXA), cefoxitin (CFX), amoxicillin/clavulanic acid (AMC), and ampicillin (AMP), fluoroquinolones: Ciprofloxacin (CIP), moxifloxacin (MOX), fusidanes: fusidic acid (FUS), lincosamides: clindamycin (CLI), macrolides: erythromycin (ERY), aminoglycosides: gentamycin (GEN), tetracyclines: tetracycline (TET), ansamycins: rifampicin (RIF), glycopeptide: teicoplanin (TEI), and Folate pathway inhibitors: trimethoprim/sulfamethoxazole (SXT).

–, the isolates were negative for the tested antibiotics and the MAR index.

### 3.4 Molecular typing of *Staphylococcus aureus* isolates

**Figure 2:**
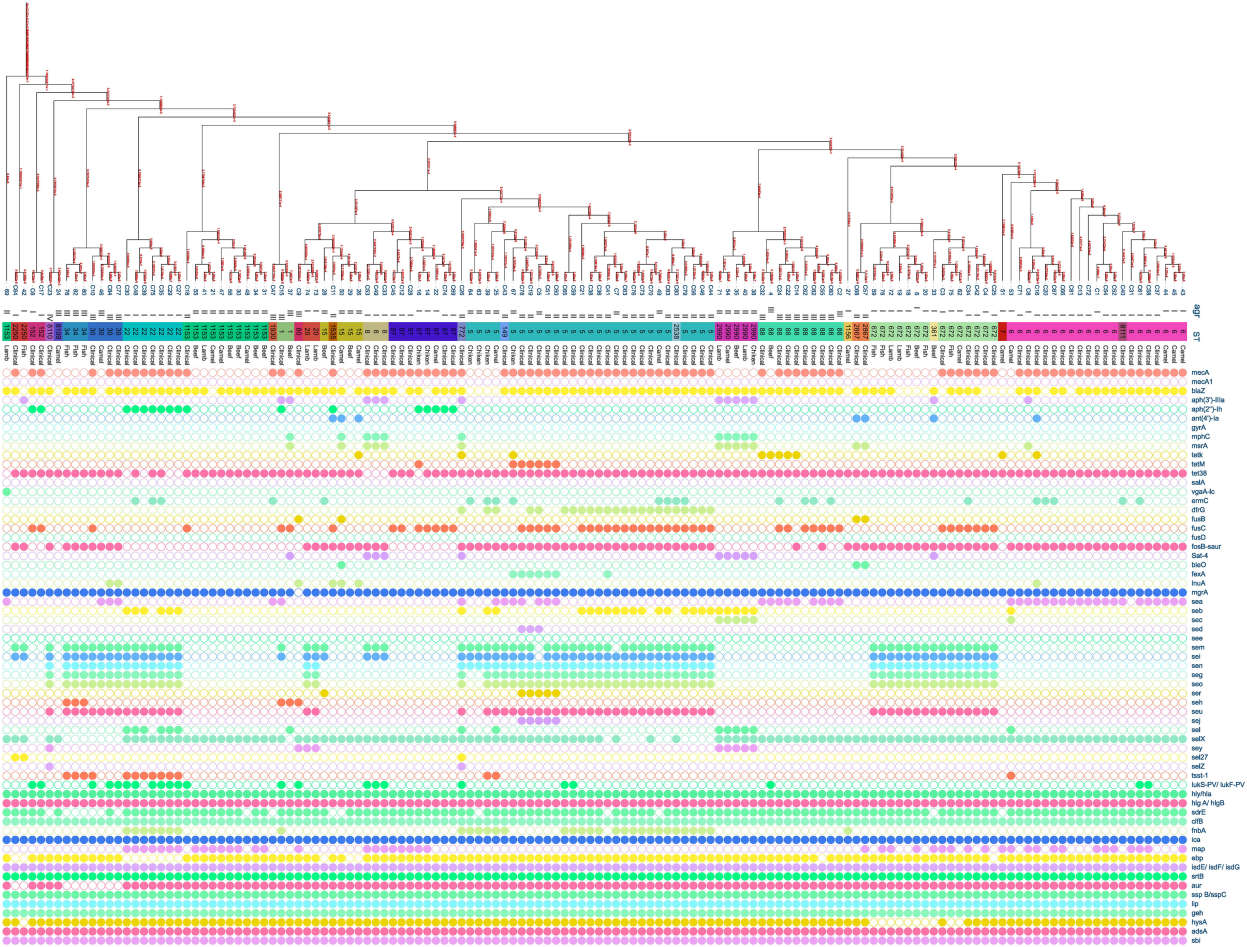
Distribution of antibiotic resistance genes, enterotoxin genes, and virulence genes plotted against core genome phylogeny of *S. aureus* isolates based on single nucleotide polymorphisms (SNPs). The *agr*, sequence types, and sources of the isolates were aligned with the tree. The fully colored circle indicates the presence of the target gene. White circles indicate the absence of investigated genes.

Multiple *S. aureus* clones were found in both patients and meat, with clonal complex CC5 being the most prevalent in both groups. CC5 comprised (38/80, 47.5%) clones from patients and (5/11, 45.45%) clones from meat. CC97 was the second most common clone in meat, making up (4/11, 36%) of the MRSA clones, while in patients it was only (6/80, 8%). CC361 was represented by (2/11, 18%) of the meat MRSA isolates and (5/80, 6%) of patients’ MRSA isolates. The clonal lineages of all *S. aureus* strains isolated from the patients and meat samples were identified using MLST (**Figure 2**). Accessory gene regulator (*agr*) alleles were identified in 136 isolates by WGS. *agr* I was the most prevalent (58/136; 43%), followed by *agr* II (53/136; 39%) and *agr* III (22/136; 16%). Only one clinical *S. aureus* isolate harbored *agr* IV. The most common *agr* type of *S. aureus* isolated from meat was *agr* II, whereas *agr* I was the most common *agr* type isolated from the patients (**Figure 2**).

### 3.5 Antibiotic-resistance genes in *Staphylococcus aureus* strains

**Figure 3:**
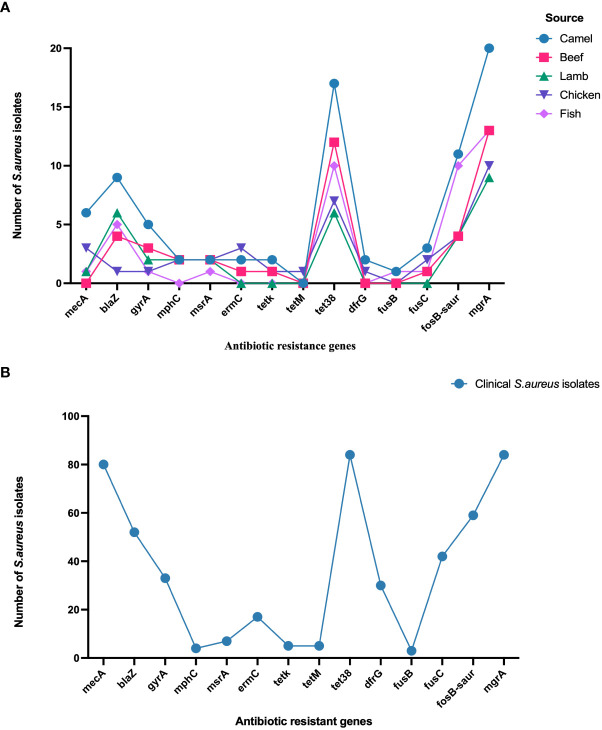
**(A)** Distribution of antibiotic resistance genes in *S. aureus* isolated from meat. **(B)**. Distribution of antibiotic resistance genes in *S. aureus* isolated from patients.

*Staphylococcus aureus* isolates from patients and meat samples demonstrated similar antimicrobial resistance gene profiles. Of the 53 meat isolates, 11 (21%) were *mec*A gene-positive and 42 (79%) were *mec*A gene-negative. Overall, the majority of *S. aureus* isolates from meat and patients contained *β* -lactam, tetracycline, and phosphonic acid resistance genes (**Figures 3A, B**). Meat isolates displayed a high prevalence of resistance genes harboring *tet*38 (69%), *bla*Z (70%), and *fos*B (58%), whereas patient isolates harbored *tet*38 (100%), *bla*Z (62%), and *fos*B (70%) (**Figures 3A, B**). A significant difference was observed between the prevalence of antibiotic resistance genes in *S. aureus* isolates from meat and patients (*p* < 0.0001). Meat isolates had multidrug resistant genes (33/53; 62.2%), specifically 100% MRSA and 52% MSSA, containing antibiotic resistance genes for three or more drug classes. All patient isolates (100%) harbored several drug resistance genes (**Table 2**).

**Table 2:** The prevalence of resistance genes among S. aureus isolated from meat and patients.

Antibiotic class	Resistance gene	The protein encoded by the gene	Camel (n = 18) (%)	Beef (n = 12) (%)	Lamb (n = 8) (%)	Chicken (n = 7) (%)	Fish (n = 8) (%)	Clinical (n = 83) (%)	Total (N = 136) (%)
**Aminoglycoside**	*aph(3’)-IIIa*	Aminoglycoside 3’-phosphotransferase	1(4.3)	3 (21.4)	2 (18.1)	1 (7.6)	1 (6.6)	6 (7.1)	14 (8.7)
	*aph(2’’)-Ih*	Aminoglycoside 2’’-phosphotransferase	1(4.3)	–	–	2 (15.3)	–	14 (16.6)	17 (10.6)
	*ant(4’)-Ia*	Aminoglycoside adenyltransferase	2 (8.6)	–	–	–	1 (6.6)	4 (4.7)	7 (4.3)
**Beta-lactam**	*mecA*	Alternative penicillin-binding protein (PBP 2a)	6 (26)	–	1 (9)	3 (23)	1 (6.6)	80 (96.3)	91 (56.8)
	*mecA1*		3 (13)	–	1 (9)	1 (7.6)	1 (6.6)	–	6 (3.7)
	*blaZ*	Beta-lactamase	9 (39.1)	4 (17.3)	6 (54.5)	1 (7.6)	5 (33.3)	52 (61.9)	77 (48.1)
**Quinolone**	*gyrA*	DNA gyrase subunit A	5 (21.7)	3 (21.4)	2 (18.1)	1 (7.6)	1 (6.6)	33 (39.2)	45 (28.1)
**Macrolide**	*mphC*	Macrolide 2’-phosphotransferase	2 (8.6)	2 (14)	2 (18.1)	2 (15.3)	–	4 (4.7)	12 (7.5)
**Macrolide/streptogramin**	*msrA*	Peptide methionine sulfoxide reductase	2 (8.6)	2 (14)	2 (18.1)	2 (15.3)	1 (6.6)	7 (8.3)	16 (10)
**Tetracycline**	*tetK*	Tetracycline resistance protein	2 (8.6)	1 (7.1)	–	1 (7.6)	–	5 (6)	9 (5.6)
	*tetK*		–	–	–	1 (7.6)	–	5 (6)	6 (3.7)
	*tet38*	Tetracycline efflux MFS transporter	17 (73.9)	12 (85.7)	6 (54.5)	7 (53.8)	10 (66.6)	83 (100)	136 (85)
**Streptogramin/lincosamide/pleuromutilin**	*salA*	Iron-sulfur cluster carrier protein	3 (13)	2 (14)	1 (9)	1 (7.6)	1 (6.6)	–	6 (3.7)
	*vgaA-lc*	ABC transporter	1(4.3)	–	1 (9)	–	1 (6.6)	–	3 (1.8)
**Streptogramin/lincosamide/macrolide**	*ermC*	rRNA adenine N-6-methyltransferase	2 (8.6)	1 (7.1)	–	3 (23)	–	17 (20.2)	23 (14.3)
**Diaminopyrimidine**	*dfrG*	Dihydrofolate reductase	2 (8.6)	–	–	1 (7.6)	–	30 (35.7)	33 (20.6)
**Fusidane**	*fusB*	2-domain zinc-binding protein	1(4.3)	–	–	–	1 (6.6)	3 (3.6)	5 (3.1)
	*fusC*		3 (13)	1 (7.1)	–	2 (15.3)	1 (6.6)	42 (50)	49 (30.6)
**Phosphonic acid**	*fosB-saur*	Metallothiol transferase	11 (47.8)	4 (17.3)	4 (36.2)	4 (38.4)	10 (66.6)	59 (70.2)	92 (57.5)
**Nucleoside**	*Sat-4*	Streptothricin N-acetyltransferase and streptothricin	1(4.3)	3 (21.4)	2 (18.1)	1 (7.6)	–	5 (6)	12 (7.5)
**Glycopeptide**	*bleO*	Bleomycin resistant proteins	1(4.3)	–	–	–	1 (6.6)	2 (2.3)	4 (2.5)
**Phenicol**	*fexA*	Chloramphenicol/florfenicol exporter	–	–	–	1 (7.6)	–	6 (7.1)	7 (4.3)
**Lincosamide**	*lnuA*	Lincosamide nucleotidyltransferase	2 (8.6)	–	–	–	–	4 (4.7)	6 (3.7)

N, total number of S. aureusS. aureus isolates in this study; n, number of S. aureusS. aureus isolates positive for a gene profile; –, no isolates positive for a gene. The numbers in parentheses indicate percentages.

### 3.6 Prevalence of enterotoxin genes in *Staphylococcus aureus* strains

**Figure 4:**
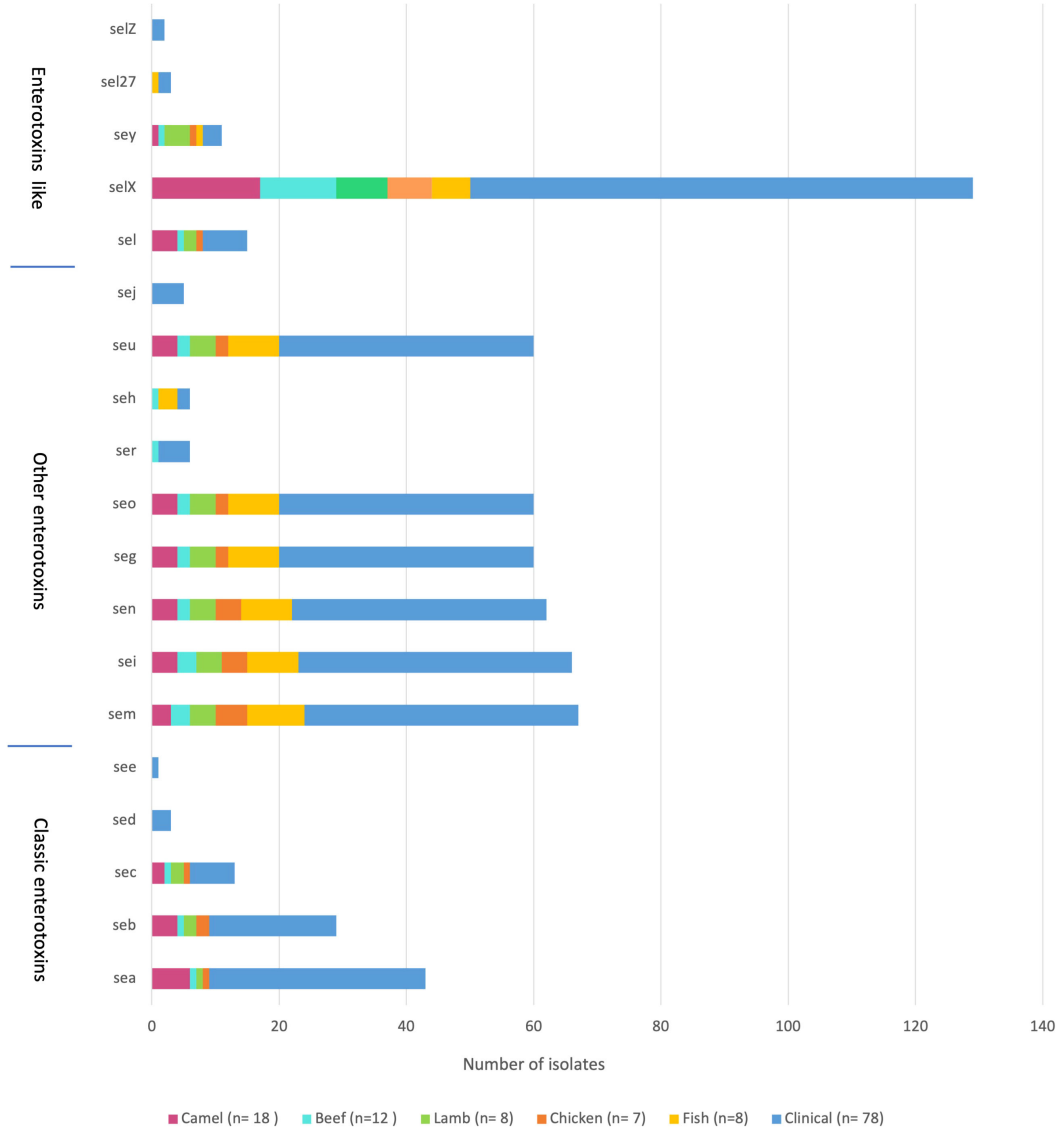
Distribution of enterotoxin genes in *S. aureus* isolated from meat and patients.

**Figure 5:**
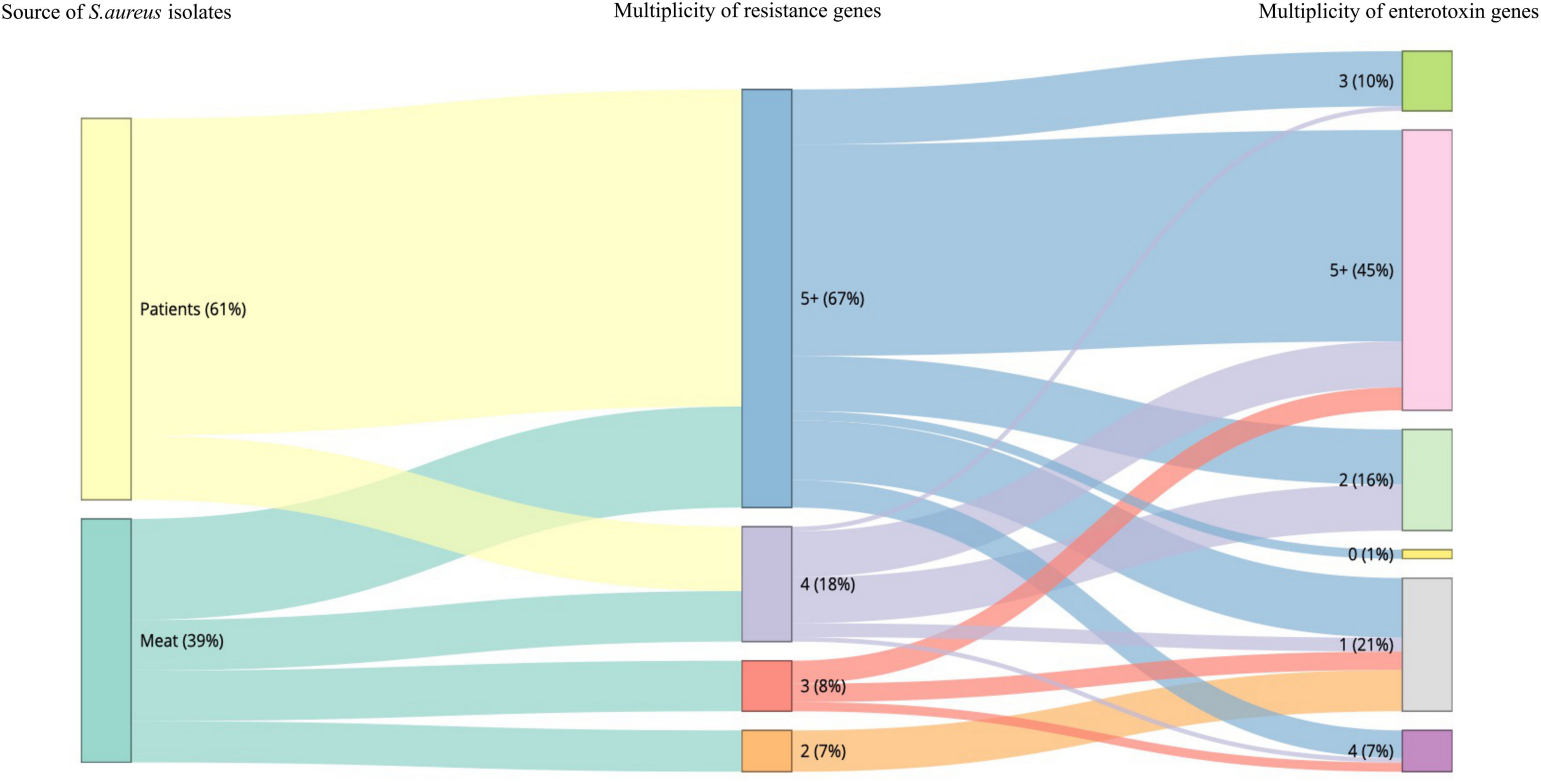
Snakey diagram illustrating the multiplicity of resistance and enterotoxin genes present in *S. aureus* isolates from both meat samples and patients.

The sequencing results revealed 131 *S. aureus* isolates harboring different enterotoxin genotypes (**Figure 4**). The 18 enterotoxin genotypes detected in this study included the classic enterotoxin genes (*sea*, *seb*, *sec, sed*, and *see*), enterotoxin-like genes (*sel*, *selX*, *sel27, selz*, and *sey*), and other enterotoxin genes (*sej, seh, ser, seu, seg, seo, sen, sem*, and *sei*). MSSA and MRSA isolates from meat and patients harbored enterotoxin genes, which varied from 0 to 12. The enterotoxin-like gene *selX* was detected in almost all *S. aureus* isolates (129/136; 94.8%), followed by *the sem* and *sei* genes (66/136; 48.5%) and *sen* (62/136; 45.5%). The classic genes *see* and *sed* were absent from meat isolates but were present in three patient isolates. All other enterotoxin genes (*seu, seg, seo, sen, sem*, and *sei*) were observed more frequently in all the isolates. The prevalence of different enterotoxin genes in patient isolates was higher than that in the meat isolates (*p* =0.0239). A large proportion of the *S. aureus* isolates (104/136, 76.4%) harbored more than one enterotoxin gene (**Table 3**). The highest prevalence was found in isolates with the genotype *selX*, *sea* (23%) followed by genotype *selX* (22%). More than 67% of the meat and patient isolates had more than five different antibiotic resistance genes and more than 45% of these isolates had more than five enterotoxins (**Figure 5**).

**Table 3:** Enterotoxin gene patterns of Staphylococcus aureus.

Pattern	Enterotoxins genes profiles	No. of meat *S. aureusS. aureus* isolates with the genotype (%)	No. of clinical *S. aureusS. aureus* isolates with the genotype (%)	No. of total *S. aureusS. aureus* isolates with the genotype (%)
P1	*selX*	18 (33.9)	11 (13.2)	29 (21.3)
P2	*selX, sem, sei, sen, seg, seo, seu*	10 (18.8)	10 (12)	20 (14.7)
P3	*selX, sem, sei, sen, seg, seo, seu, sea, seb, sel*	1 (1.8)	0 (0)	1 (0.7)
P4	*selX, sem, sei, ser*	1 (1.8)	0 (0)	1 (0.7)
P5	*sec, selX, seb, sel, sey*	5 (9.4)	0 (0)	5 (3.6)
P6	*selX, seh*	1 (1.8)	0 (0)	1 (0.7)
P7	*selX, sem, sei, sen, seg, seo, seu, seb*	2 (3.7)	12 (14.4)	14 (10.2)
P8	*sem, sei, sen, seg, seo, seu, sea*	1 (1.8)	2 (2.4)	3 (2.2)
P9	*selX, sea, seb, sel*	1 (1.8)	0 (0)	1 (0.7)
P10	*selX, sem, sei, sen*	2 (3.7)	0 (0)	2 (1.4)
P11	*selX, sem, sei, sen, seg, seo, seu, sea*	1 (1.8)	1 (1.2)	2 (1.4)
P12	*selX, sem, sei, sen, seg, seo, seu, sey*	2 (3.7)	0 (0)	2 (1.4)
P13	*sem, sei, sen, seg, seo, seu, seh*	3 (5.6)	0 (0)	3 (2.2)
P14	*selX, sen, seg, seo, seu, sea, sej, ser, sed*	0 (0)	1 (1.2)	1 (0.7)
P15	*selX, sey, seh*	0 (0)	1 (1.2)	1 (0.7)
P16	*sem, sei, sen,seg, seo, seu*	0 (0)	1 (1.2)	1 (0.7)
P17	*selX, sem, sei, sea, seh*	0 (0)	1 (1.2)	1 (0.7)
P18	*selX, sem, sei, sen, seg, seo, seu, sej, ser, sed*	0 (0)	1 (1.2)	1 (0.7)
P19	*selX, sem, sei, sen, seg, seo, seu, sey, selZ*	0 (0)	1 (1.2)	1 (0.7)
P20	*sec, selX, sem, sei, sen, seg, seo, seu, sea, seb, sel, sel27, selZ*	0 (0)	1 (1.2)	1 (0.7)
P21	*sec, selX, sem, sei, sen, seg, seo, seu, seb, sel*	0 (0)	6 (7.2)	6 (4.4)
P22	*selX, sem, sei, sen, seg, seo, seu, seb, sel, sen*	0 (0)	1 (1.2)	1 (0.7)
P23	*selX, sem, sei*	0 (0)	3 (3.6)	3 (2.2)
P24	*selX, sem, sei, sen, seg, seo, seu, sea, sej, ser*	0 (0)	2 (2.4)	2 (1.4)
P25	*selX, sea*	5 (9.4)	25 (30.1)	30 (22)
P26	*selX, sei, sen, seg, seo, seu, seb*	0 (0)	1 (1.2)	1 (0.7)

### 3.7 Prevalence of virulence genes in *Staphylococcus aureus* strains

Toxin genes were distributed in a similar pattern in *S. aureus* isolates (**Table 4**). The virulence genes *cap*, *hly/hla*, *sbi*, *geh*, *hlgA/hlgB*, *esaA/esaB*, *essA/essB*, *esxA*, *ica, lip, hld, adsA, sspB/sspC, isdE/isdF/isdG*, and *srtB* were detected in all isolates. Almost all isolates harbored *sspA*, *isdB*/*isdC*/*isdD* (134/136; 99%), *hlgC* (133/136; 98%), *isdA* (132/136; 97%), *aur* (129/136; 95%), and *ebp* (127/136; 93%) genes. Other virulence genes were also prevalent among the isolates, including *hysA* (118/136; 87%), *sdrE* (116/136; 85%), *sdrC* (113/136; 83%), *sdrD* (112/136; 82%), *esaC and esxB* (107/136; 79%), *essC* (106/136; 78%), and *clfB* (92/136; 68%). Other virulence genes were also present: *map* (46/136; 34%), *fnbA* (32/136; 24%), *clfA* (27/136; 20%), *pvl* (lukS-PV/lukF-PV) (24/136; 18%), *sell* (15/136; 11%), and *tst-1* (14/136; 10%). Six isolates of *S. aureus* carrying the toxin gene *tst-1* were identified in meat, including two camels, one chicken, three fish, and eight from patients. Furthermore, a single *S. aureus* isolate from beef and 23 isolates obtained from patients harbored the *pvl* genes (*lukS-PV/lukF-PV*). A significant difference in the distribution of virulence genes was observed between *S. aureus* isolates from meat and patients (p < 0.0001).

**Table 4:** Distribution of virulence-associated gene among S. aureus isolate.

Gene product	Virulence gene	Camel (n= 18) (%)	Beef (n=12) (%)	Lamb (n= 8) (%)	Chicken (n= 7) (%)	Fish (n=8) (%)	Clinical (n= 83) (%)	Total (N= 136) (%)
Toxic shock syndrome toxin 1	*tsst-1*	2 (1.1)	–	–	1(14.2)	3 (37.5)	8 (9.6)	14 (10.2)
Panton–Valentine Leukocidin	*lukS-PV*	–	1 (8.3)	–	–	–	23 (27.7)	24 (17.6)
Panton–Valentine Leukocidin	*lukF-PV*	–	1 (8.3)	–	–	–	23 (27.7)	24 (17.6)
Capsular polysaccharide synthesis enzyme	*cap*	18 (100)	12 (100)	8 (100)	7(100)	8 (100)	83(100)	136 (100)
Alpha-Hemolysin precursor	*hly/hla*	18 (100)	12 (100)	8 (100)	7(100)	8 (100)	83(100)	136 (100)
Fibronectin-binding protein A	*fnbA*	3 (16.6)	–	–	3 (42.8)	–	26 (31.3)	32 (23.5)
Cell surface elastin binding protein	*ebp*	18 (100)	12 (100)	8 (100)	7(100)	8 (100)	74 (89.1)	127 (93.3)
Clumping factor, A fibrinogen-binding protein	*clfA*	5 (27.7)	7 (58.3)	2 (25)	2 (28.5)	–	11 (13.2)	27 (19.8)
	*clfB*	11 (61.1)	8 (66.6)	2 (25)	6 (85.7)	6 (75)	59 (71)	92 (67.6)
IgG-binding protein	*sbi*	18 (100)	12 (100)	8 (100)	7(100)	8 (100)	83(100)	136 (100)
Gamma-hemolysin chain II precursor	*hlgA*	18 (100)	12 (100)	8 (100)	7(100)	8 (100)	83(100)	136 (100)
Ser-Asp rich fibrinogen-binding bone sialoprotein-binding protein	*sdrE*	12 (66.6)	9 (75)	8 (100)	6 (85.7)	5 (62.5)	76 (91.5)	116 (85.2)
Type VII secretion system protein	*esaA*	18 (100)	12 (100)	8 (100)	7(100)	8 (100)	83(100)	136 (100)
	*essA*	18 (100)	12 (100)	8 (100)	7(100)	8 (100)	83(100)	136 (100)
	*esxA*	18 (100)	12 (100)	8 (100)	7(100)	8 (100)	83(100)	136 (100)
Zinc metalloproteinase aureolysin	*aur*	17 (94.4)	12 (100)	8 (100)	7(100)	5 (62.5)	80 (96.3)	129 (94.8)
Triacylglycerol lipase precursor	*lip*	18 (100)	12 (100)	8 (100)	7(100)	8 (100)	83(100)	136 (100)
Delta-hemolysin	*hld*	18 (100)	12 (100)	8 (100)	7(100)	8 (100)	83(100)	136 (100)
Adenosine synthase A	*adsA*	18 (100)	12 (100)	8 (100)	7(100)	8 (100)	83(100)	136 (100)
Serine protease; V8 protease; glutamyl endopeptidase	*sspB*	18 (100)	12 (100)	8 (100)	7(100)	8 (100)	83(100)	136 (100)
Iron-regulated surface determinant protein	*isd A*	18 (100)	12 (100)	8 (100)	7(100)	8 (100)	79 (95.1)	132 (97)
	*isd E*	18 (100)	12 (100)	8 (100)	7(100)	8 (100)	83(100)	136 (100)
NPQTN specific sortase B	*srtB*	18 (100)	12 (100)	8 (100)	7(100)	8 (100)	83(100)	136 (100)
Hyaluronate lyase precursor	*hysA*	17 (94.4)	11(91.6)	5 (62.5)	7(100)	3 (37.5)	75 (90.3)	118 (86.7)
Extracellular proteins Map	*map*	5 (27.7)	7 (58.3)	2 (25)	2 (28.5)	2 (25)	28 (33.7)	46 (33.8)

n, total number of isolates from the type of sample; N, total number of S. aureus isolates; –, no isolates positive for this virulence gene the n, number of total isolates from the type of sample; N, total number of S. aureus isolates; –, no isolates positive for this virulence gene.

The prevailing resistance gene patterns observed among MRSA isolates derived from both meat sources and human patients encompass *mecA, aph(2’’)-Ih, fusC*, and *tet38* which were identified in three meat samples associated with the MRSA Clonal Complex 97 (CC97). In the patients, they were present in MRSA isolates belonging to CC97, CC152, and CC153. In the context of virulence genes, the distribution patterns of genes exhibited nearly uniform prevalence across various clonal complexes.

## 4 Discussion

In this study, *S. aureus* isolates were found in 21.2% of retail meat samples. Similarly, several previous studies have reported comparable levels of *S. aureus* contamination in raw meat: 27.8% prevalence in the United States (Thapaliya et al., 2017), 24.5% in various African countries (Thwala et al., 2021), 23.8% in Iran (Safarpoor Dehkordi et al., 2017), 35% in China, 33.3% in the Netherlands (van Loo et al., 2007), and 22% in Bangladesh (Pu et al., 2009). Similarly, several earlier investigations have reported comparable levels of *S. aureus* contamination in raw meat: 27.8% prevalence in the United States (Thapaliya et al., 2017), 24.5% in various African countries (Thwala et al., 2021), 23.8% in Iran (Safarpoor Dehkordi et al., 2017), 35% in China, 33.3% in the Netherlands (van Loo et al., 2007), and 22% in Bangladesh (Pu et al., 2009). Various factors, such as hygiene practices, storage conditions, and the use of antibiotics in animal husbandry can differ between regions and impact the presence of *S. aureus* in meat (Dorjgochoo et al., 2023). These regional variations are essential for understanding and addressing the food safety concerns associated with *S. aureus* contamination.

In addition, 136 *S. aureus* isolates had diverse genetic backgrounds as demonstrated by MLST and *agr* typing. The most prevalent *agr* type in patient isolates in the present study was *agr* I. This finding aligns with previous reports indicating the predominance of *agr* I among MRSA isolates associated with clinical infections in Riyadh (54.21% and 42.4%, respectively) (Monecke et al., 2012; Senok et al., 2019). In contrast, the predominant *agr* type in meat in this study was *agr* II. A previous study reported the prevalence of *agr* types I, II, and III in *S. aureus* isolated from retail meat in Riyadh, all of which were evenly distributed across isolates (Raji et al., 2016). Although *agr* IV has previously been identified in other clinical *S. aureus* isolates, only one clinical isolate in the present study harbored the same *agr* type (Monecke et al., 2012; Senok et al., 2019). The incompatibility of *agr* types over time indicates the flexibility of *S. aureus* isolates for adaptation. The clinical *S. aureus* isolates harboring *agr* IV in this study shared the same clonal lineage (CC121) as those from retail meat, carrying the same *agr* type, thereby demonstrating genetic relatedness (Raji et al., 2016).

The high prevalence of antibiotic resistance genes in both patient and meat isolates is concerning because it underscores the potential for the transmission of resistance traits from the food supply to the clinical setting. *tet*38, which encodes tetracycline resistance, was found in 100% and 69% of patients and meat isolates, respectively. A previous study reported a *tet*K prevalence of 4% among *S. aureus* isolates from retail meat (Raji et al., 2016). Additionally, in a hospital in Riyadh, *S. aureus* isolates had *tet*K and *tet*M prevalence rates of 10.2% and 20.5%, respectively (Monecke et al., 2012). The increase in the prevalence of *tet* gene may indicate the excessive use of tetracyclines over the past few years. Moreover, *β* -lactam resistance genes, including *bla*Z, were highly prevalent in 70% and 62% of meat and patients, respectively. Comparatively, these rates slightly decreased compared to previous studies in Saudi Arabia, where isolates from retail meat in Riyadh had a *blaZ* prevalence rate of 88% (Raji et al., 2016) and those from hospitals in Riyadh were 93.46% (Monecke et al., 2012). Furthermore, the gene encoding phosphonic acid resistance, *fos*B, had prevalence rates of 70% and 58% in the patient and meat isolates in the present study, respectively. This is consistent with a previous study on retail meat, wherein 52% of *S. aureus* isolates harbored *fos*B (Raji et al., 2016). The high prevalence of antibiotic -resistance genes is most likely a result of the overuse of *β*-lactams and phosphonic acid antibiotics to treat *S. aureus* infections in hospitals and livestock farms.

The distribution of antibiotic resistance genes among MRSA strains in a tertiary care facility in Riyadh was as follows: *erm*C (28.8%), *msr*A (10.4%), *aph*A3 and *sat* (18.4%), *fus*C (43.2%), *tet*K (17.6%), *tet*M (7.2%), and *fos*B (56.8%) (Senok et al., 2019). According to Snoussi et al. (2023), the genes associated with antimicrobial resistance in *S. aureus* isolates included *fus*C (50%), *fos*B, *tet*K, *tet*45, *sat*-4, *aph*(3′)-IIIa (12%), *aac*(6′)*-aph*(2″), and *erm*C (25%). In the present study, resistance genes in meat isolates included *aph*(3’)-IIIa (15%), *gyr*A (23%), *tet*K (7%), *tet*M (2%), *erm*C (9%), *fos*B (62%), *msr*A, *fus*C, and *sat*-4 (13%), whereas those in patient isolates included *aph*(3’)-IIIa (7%), *gyr*A (40%), *msr*A (8%), *erm*C (20%), *fus*C (51%), *fos*B (71%), *tet*K, and *sat*-4 (6%). Although some antibiotic resistance genes had higher prevalence levels in meat isolates, such as *aph*(3’)-IIIa, *tet*K, *msr*A, and *sat*-4, patient isolates harbored more antimicrobial resistance genes. This might due to the environment in which patient isolates are genetically adapted to thrive and flourish in hospitals. The fact that all patient isolates harbored multidrug-resistant genes is particularly alarming, highlighting the importance of prudent antibiotic use in healthcare. Furthermore, the presence of multidrug-resistant *S. aureus* strains in meat isolates raises concerns regarding the role of foodborne transmission in the dissemination of antibiotic resistance, highlighting the importance of surveillance in food production and distribution.

The high prevalence of some antibiotic genes in this study, such as *fos*B, in MRSA isolates raises concerns regarding the potential compromise of fosfomycin as a last-resort therapy for staphylococcal infections. Fosfomycin is used to treat MRSA and MDR infections because it can penetrate biofilms and exert intracellular bactericidal activity because of its low molecular weight (Chen et al., 2022). Fosfomycin is often used in combination with other antibiotics to reach targets more efficiently, thereby improving the therapeutic effects of the combined antibiotics (Chen et al., 2022). The high prevalence of *fos*B gene, highlights the risk of emerging resistance, which affects the efficacy of fosfomycin in the treatment MRSA infections. In the present study, the MAR index values (0.2–0.5) of most MDR *S. aureus* strains revealed multiple resistance patterns, suggesting that the *S. aureus* strains were derived from high-risk contamination (Krumperman, 1983). This result emphasizes the potential public health implications of antimicrobial resistance in *S. aureus* isolates.

*Staphylococcus aureus* enterotoxins are gastrointestinal exotoxins that can cause SFP when consumed in certain amounts (Argudín et al., 2010). Staphylococcal enterotoxin A (*sea*) is the most frequently identified cause of SFP, and classic enterotoxins (*sea, seb, sec, sed, and see*) are associated with the most reported food poisoning outbreaks (Cha et al., 2006). In Kuwait, a study of 200 isolates obtained from food handlers in various restaurants with suspected SFP revealed that the majority of isolates were resistant to various antibacterial agents, and that 71% of the isolates harbored genes for SEs, with *sei* being the most prevalent (Udo et al., 2009). In the present study, the enterotoxin-like gene *sel*X was found in 95% of the patient isolates, followed by the *sem* and *sei* genes, and *sen*. Meat isolates exhibited comparable prevalence rates. These rates differ depending on the source of isolation and area. For instance, in Riyadh, a previous study isolated *S. aureus* from dairy products and identified 14 enterotoxin genes, of which *seh* had the highest ratio (51%). Furthermore, classic enterotoxins have a lower prevalence, except for the *see* gene (27.5%) (Alghizzi and Shami, 2021). In another study, the prevalence of *seh* gene in *S. aureus* isolates from processed meat was the highest at 49% (Alghizzi et al., 2021). In 2014, 165 *S. aureus* isolates were obtained from food handlers in Makkah, and PCR analysis of classic SEs genes revealed that *sea* was the most prevalent enterotoxin in MRSA (36%) and MSSA (30%) isolates (Ahmed and Mashat, 2014). Similarly, the most prevalent classic enterotoxin in *S. aureus* isolates in the present study was *sea*. The prevalence of classic SEs genes in MRSA isolates from patients in Riyadh in 2012 was < 9% (Monecke et al., 2012). In the early 2000s, 129 *S. aureus* isolates were identified from food handlers in Makkah during the Hajj season, and *sec* and *sea* were observed in 15.5% and 12.4% of the *S. aureus* isolates, respectively (Dablool and Al-Ghamdi, 2011). In the present study, *sec* was detected in 10% of the *S. aureus* isolates, and the prevalence of the *sea* gene was 32%. The high compatibility of enterotoxin genotypes in *S. aureus* isolates from meat and patients indicates a strong genomic association between the isolates from both sources. Furthermore, the higher prevalence of enterotoxin genes in the patient isolates suggests their role in the clinical manifestations of *S. aureus* infections, potentially leading to more severe symptoms.

Every toxin-encoding gene is equally important; the more genes an organism possesses, the more virulent it becomes (Tam and Torres, 2019). In Saudi Arabia, information regarding the prevalence and molecular characteristics of these virulence genes is limited. However, our analysis revealed a consistent distribution pattern of virulence genes across *S. aureus* isolates from meat samples and patients, indicating a common genetic structure among pathogens from both sources. Notably, key virulence genes, including *cap, hly/hla, sbi, geh, hlg*A*/hlg*B*, esa*A*/esa*B*, ess*A*/ess*B*, esx*A*, ica, lip, hld, ads*A*, ssp*B*/ssp*C*, isd*E*/isd*F*/isd*G*, and srt*B were present in all *S. aureus* isolates. This consistency suggests that these genes play essential roles in the pathogenesis of *S. aureus* from various sources. Although a significant difference in the distribution of virulence genes was observed between *S. aureus* isolates from meat samples and patients (p < 0.0001), it is essential to note the different sample sizes in each group. Nevertheless, this significance underscores the robust association between virulence gene distribution and the source of *S. aureus* isolates.

*S. aureus* produces the cytotoxin PVL, which destroys leukocytes and results in tissue necrosis (Otto, 2014). Previously, *pvl* gene was not abundant, except in some cases, and was often associated with CA-MRSA strains (Alghizzi and Shami, 2021; Alghizzi et al., 2021). For example, this gene was not detected in 50 *S. aureus* isolates obtained from patients in Makkah in 2017 (Abulreesh et al., 2017). In addition, it is absent in *S. aureus* isolated from dairy products, milk, and processed foods in Riyadh (Alghizzi and Shami, 2021; Alghizzi et al., 2021). In this study, 28% of *S. aureus* isolates from patients harbored *pvl*, and only one *S. aureus* isolate from beef that belonged to LA-MRSA CC361 harbored this gene. A previous study of 125 MRSA isolates linked to clinical infections in Riyadh reported a *pvl* prevalence of 30% (Senok et al., 2019). The high prevalence of *pvl*-carrying *S. aureus* clones in hospitals, which is known for its association with CA-MRSA, suggests that CA-MRSA isolates outnumber HA-MRSA in hospitals and that there is an over-time shift in the distribution of MRSA types.

Toxic shock syndrome (TSS) exotoxin is a superantigen that is generally resistant to heat and proteolysis and induces T cell-dependent shock syndrome with significant mortality by boosting the excessive release of cytokines (Dinges et al., 2000). Although *S. aureus* isolated from meat had a slightly higher prevalence of the *tst* gene in this study, it did not vary greatly from that of *S. aureus* isolated from patients. These numbers are slightly lower than the national *tst* gene prevalence rates, which range from 11.2% to 13.2% in human infections (Alkharsah et al., 2018; Senok et al., 2019). Evaluation of the frequency of virulence genes in *S. aureus* strains serves as a fundamental basis for formulating effective infection control strategies and anti-virulence interventions to suppress *S. aureus* with hypervirulent genes (Dinges et al., 2000). A limitation of this study was the small sample size, which was determined by the city’s landscape within a confined geographical area. Moreover, this study was constrained by the comparison of MRSA isolates from symptomatic humans with *S. aureus* and MRSA isolated from meat products. It is important to note, however, that our findings align with previous research conducted in this region (Raji et al., 2016; Aljeldah, 2020; Alghizzi et al., 2021).

The observed correlation in genetic lineages, antibiotic resistance profiles, enterotoxin genes, and virulence genes between the two sources of *S. aureus* has significant implications for public health. Further research is recommended to explore the genetic factors involved in *S. aureus* pathogenicity and their potential impact on clinical outcomes and food safety. Additionally, protocols to monitor and limit the transmission of *S. aureus* between clinical and foodborne settings should be developed to mitigate the potential risks to public health.

## 5 Conclusion

Our findings provide a comprehensive exploration of the genomic properties of the clinical and foodborne *S. aureus* strains. The results strongly support the close genetic association between *S. aureus* isolates from meat and patients, revealing shared antibiotic resistance and virulence gene profiles. Notably, the detection of a diverse array of these genes in meat-derived *S. aureus*, which is highly compatible with that found in patient isolates, underscores the significant genomic relatedness observed in our study. The high prevalence of specific genes such as *sel*X, *tet*38, *bla*Z, *fos*B, and *mec*A, highlights potential challenges associated with virulence and antibiotic resistance. Our study indicates that the misuse of antibiotics may contribute to the dissemination of multidrug resistance across *S. aureus* isolates from different settings, with meat serving as a potential reservoir for MDR dissemination. In light of these findings, a comprehensive approach is essential for estimating the prevalence, prevent, and control *S. aureus* infections, thereby improving both animal and human health.

## Data availability statement

The datasets presented in this study can be found in online repositories. The names of the repository/repositories and accession number(s) can be found in the article/**Supplementary Material**.

## Ethics statement

The study protocol was approved by the Institutional Review Board (IRB) committee of King Saud Medical City, Riyadh, Saudi Arabia (H-01-R-053) and the Institutional Biosafety and Bioethics Committee of King Abdullah University of Science and Technology (22IBEC051). The studies were conducted in accordance with the local legislation and institutional requirements. The human samples used in this study were acquired from primarily isolated as part of your previous study for which ethical approval was obtained. Written informed consent for participation was not required from the participants or the participants’ legal guardians/next of kin in accordance with the national legislation and institutional requirements.

## Author contributions

DA: Conceptualization, Data curation, Formal Analysis, Investigation, Methodology, Software, Visualization, Writing – original draft, Writing – review & editing. MA: Conceptualization, Investigation, Project administration, Supervision, Validation, Visualization, Writing – review & editing. AB: Resources, Supervision, Writing – review & editing. MAla: Data curation, Formal Analysis, Funding acquisition, Resources, Software, Supervision, Writing – review & editing. MM: Investigation, Writing – review & editing. SA: Methodology, Resources, Writing – review & editing. MAls: Methodology, Resources, Writing – review & editing. TG: Funding acquisition, Resources, Supervision, Writing – review & editing. SA: Conceptualization, Funding acquisition, Project administration, Resources, Supervision, Validation, Visualization, Writing – review & editing.
