# Supplementary material for: Comparative genomic analysis of antibiotic resistance and virulence genes in Staphylococcus aureus isolates from patients and retail meat
Source: Front Cell Infect Microbiol. 2024 Jan 12;13:1339339. doi: 10.3389/fcimb.2023.1339339 (PMC10811269; doi:10.3389/fcimb.2023.1339339)
Supplement: Supplementary file 1 [file DataSheet_1.docx]

Supplementary Material

**Comparative genomic analysis of antibiotic resistance genes, toxin genes, and virulence factors profiles in *Staphylococcus aureus* isolated from patients and retail meat**

**Dalal M. Alkuraythi^1,2^, Manal M. Alkhulaifi^1^, Abdulwahab Z. Binjomah^3,4^, Mohammed Alarwi^5^, Mohammed I. Mujallad^6^, Saleh Ali Alharbi^3^, Mohammad Alshomrani^3^, Takashi Gojobori^5^, Sulaiman M. Alajel^7^**

***Correspondence:**Dalal Alkuraythi, [Dalkrithi@uj.edu.sa](mailto:Dalkrithi@uj.edu.sa)

# Supplementary Figures and Tables

## Supplementary Figures


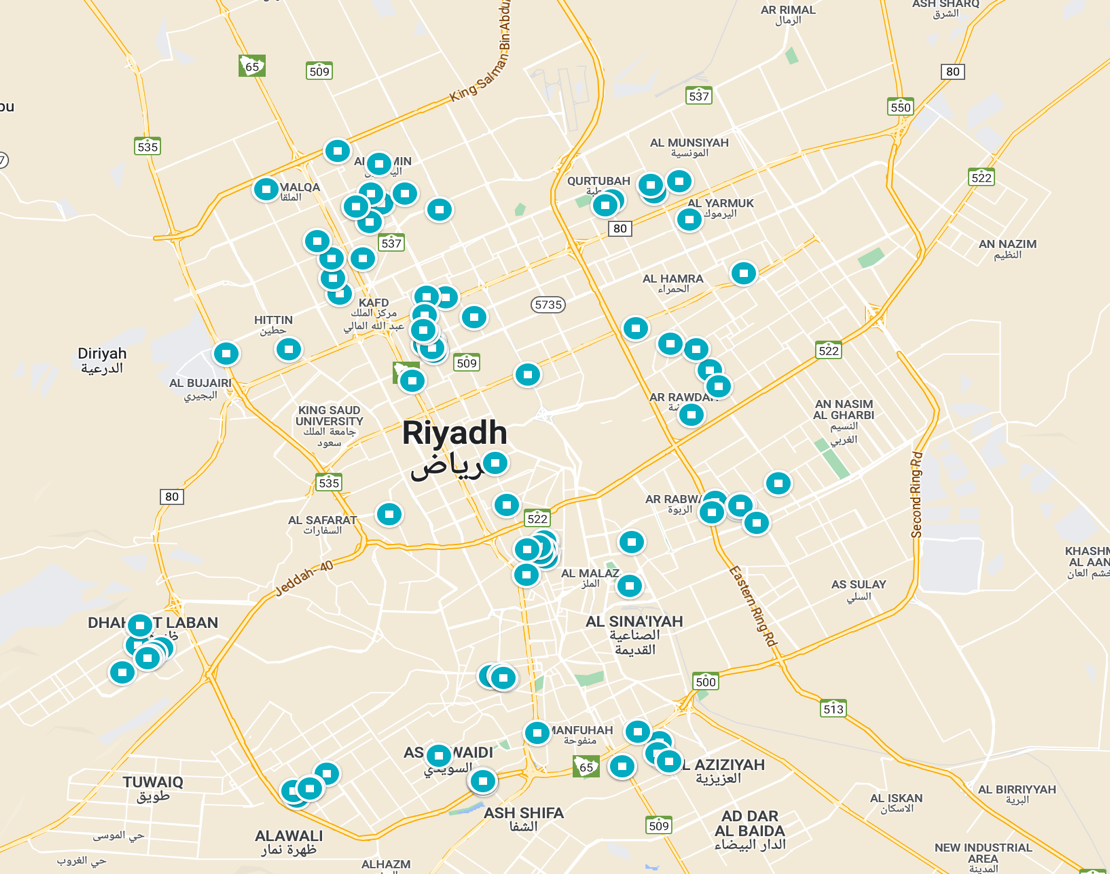


**Supplementary Figure 1.** An image of the city of Riyadh, Saudi Arabia, showing the locations of 112 Bucher shops and slaughterhouses where *S. aureus* were isolated from 250 meat different samples.


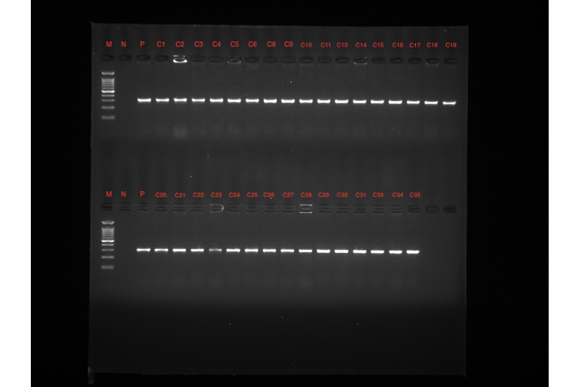


Supplementary Figure 2. PCR amplification of *mec*A gene of *S. aureus*. M: 100 bp size DNA ladder (Thermo Scientific, USA), N: Negative Control, P: Positive control (ATCC33400), Lane C1-C35: *S. aureus* isolates.


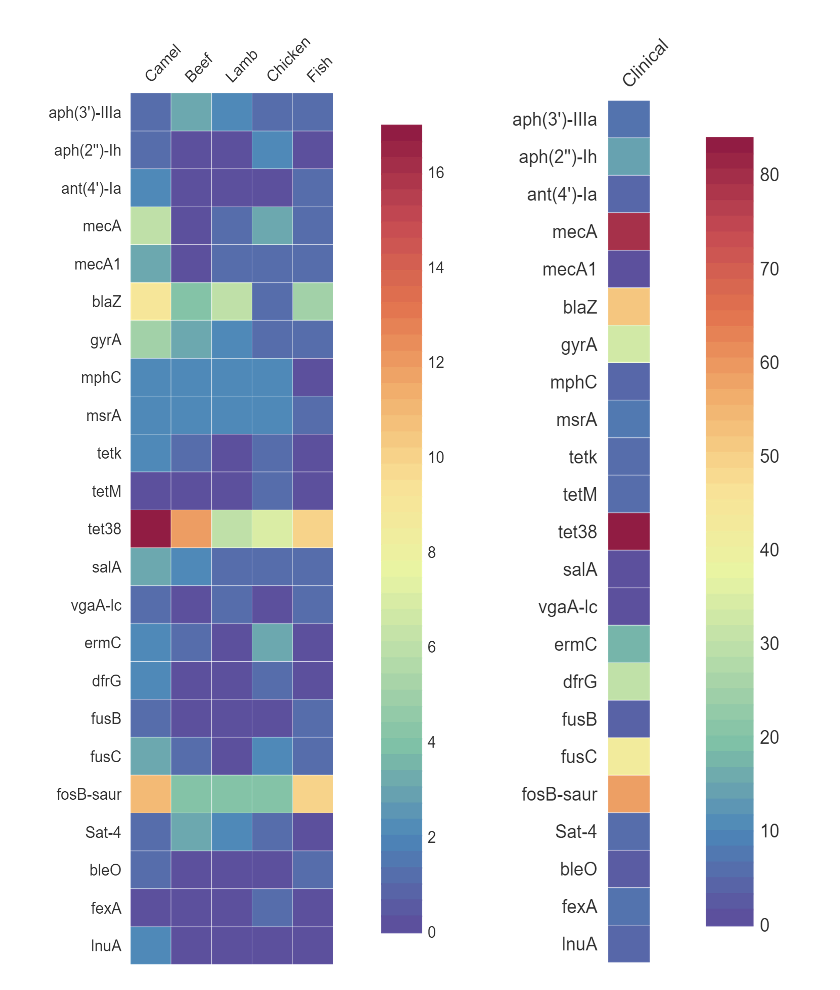


**Supplementary Figure 3.** Heatmap that shows the most common antibiotic resistance genes in *S.aureus* from different types of meat and clinical *S.aureus* isolates.

**Supplementary Figure 4.** Comparison of patients and meat *S.aureus* isolates in resistance genes, virulence genes, enterotoxins genes.

## Supplementary Tables

Supplementary Table1: Antibiotic resistance genotype profiles of *S.aureus* from meat and patients

| Resistance genotype pattern in meat *S.aureus* isolates | Resistance genes profile | No. of resistance genes patterns in *S.aureus* isolates from meat (%) |
| --- | --- | --- |
| R1 | *mecA, blaZ, fosB-Saur, tet(38), mgrA* | 3 (5.6) |
| R2 | *mecA, aph(2'')-Ih, fusC, tet(38), mgrA* | 3 (5.6) |
| R3 | *blaZ, tet(38), tet(K), mgrA* | 1 (1.8) |
| R4 | *fosB-Saur , tet(38), mgrA* | 11 (20.7) |
| R5 | *tet(38), mgrA* | 9 (16.9) |
| R6 | *dfrG, erm (C), fosB-Saur , tet (38), mgrA* | 2 (3.7) |
| R7 | *ant(4')-Ia ,blaZ, fosB-Saur,lnu(A), tet(38), tet(K), mgrA* | 1 (1.8) |
| R8 | *blaZ, erm (C), fosB-Saur, tet(38), mgrA* | 1 (1.8) |
| R9 | *ant(6)-Ia, aph(3')-IIIa, blaZ, fosB-Saur, sat4, tet(38), mgrA* | 1 (1.8) |
| R10 | *aph(3')-IIIa, blaZ, mph (C), msr(A), sat4, tet(38), mgrA* | 5 (9.4) |
| R11 | *aph(3')-IIIa, blaZ, fusC, mph (C), msr(A), sat4, tet(38), mgrA* | 1 (1.8) |
| R12 | *aph(3')-IIIa ,fosB-Saur, tet(38), mgrA* | 1 (1.8) |
| R13 | *ermC, fosB-Saur , tet(38), mgrA* | 1 (1.8) |
| R14 | *blaZ, tet(38), mgrA* | 1 (1.8) |
| R15 | *mecA, blaZ, dfrG, erm (C),fosB-Saur, fusC, tet(38), mgrA* | 1 (1.8) |
| R16 | *ant(4')-Ia , blaZ, bleO, fosB-Saur,fusB, lnu(A), mph (C), msr(A),tet(38), mgrA* | 1 (1.8) |
| R17 | *tet(38), tet(K), mgrA* | 1 (1.8) |
| R18 | *blaZ, fosB-Saur, tet(38), mgrA* | 5 (9.4) |
| R19 | *mecA, fosB-Saur, fusC, tet(38), mgrA* | 2 (3.7) |
| R20 | *mecA, erm (B), fexA ,fosB-Saur, tet(38), tet(K), tet(M), mgrA* | 1 (1.8) |
| R21 | *mecA, tet(38),vga(A)-LC, mgrA* | 1 (1.8) |
| Resistance genotype pattern in clinical *S.aureus* isolates | Resistance genes profile | No. of resistance genes patterns in *S.aureus* isolates from patients |
| P1 | *mecA, blaZ, fosB-Saur, tet(38), mgrA* | 2 (2.4) |
| P2 | *mecA, blaZ, fusC, tet(38), mgrA* | 1 (1.2) |
| P3 | *mecA, fosB-Saur ,fusC, tet(38), mgrA* | 4 (4.8) |
| P4 | *mecA, blaZ, dfrC, fexA, fosB-Saur, fusC, tet(38), tet(M), mgrA* | 5 (6) |
| P5 | *mecA, aph(2'')-Ih, fusC, tet(38), mgrA* | 5 (6) |
| P6 | *mecA, blaZ, dfrG, fosB-Saur, fusC, tet(38), mgrA* | 11 (13.2) |
| P7 | *mecA, ant(6)-Ia, aph(3')-IIIa, fosB-Saur, msr(A), sat4, tet(38), mgrA* | 1 (1.2) |
| P8 | *mecA, blaZ, fusB, tet(38), mgrA* | 1 (1.2) |
| P9 | *mecA, ant(4')-Ia, aph(2'')-Ih, blaZ, fosB-Saur ,fusC, lnu(A), tet(38), mgrA* | 1 (1.2) |
| P10 | *mecA, fusC, tet(38), mgrA* | 2 (2.4) |
| P11 | *mecA, fosB-Saur, tet(38), mgrA* | 10 (12) |
| P12 | *mecA, blaZ, fosB-Saur, tet(38), tet(K), mgrA* | 1 (1.2) |
| P13 | *mecA, aph(2'')-Ih, aph(3')-IIIa, blaZ, fusC, tet(38), tet(L), mgrA* | 1 (1.2) |
| P14 | *mecA, ant(4')-Ia, blaZ , ermC, fosB-Saur, lnu(A), tet(38), tet(K), mgrA* | 1 (1.2) |
| P15 | *mecA, ermC, fosB-Saur, tet(38), mgrA* | 4 (4.8) |
| P16 | *mecA, blaZ, fusC, tet(38), tet(K), mgrA* | 1 (1.2) |
| P17 | *mecA, blaZ, erm(C), fusC, tet(38), tet(K), mgrA* | 1 (1.2) |
| P18 | *mecA, aph(3')-IIIa, blaZ, dfrG, fosB-Saur, mph (C), msr(A), sat4, tet(38), tet (K), mgrA* | 1 (1.2) |
| P19 | *mecA, aph(2'')-Ih, blaZ, dfrC, tet(38), mgrA* | 4 (4.8) |
| P20 | *mecA, tet(38), mgrA* | 1 (1.2) |
| P21 | *mecA, blaZ, tet(38), tet(K), mgrA* | 1 (1.2) |
| P22 | *mecA, aph(3')-IIIa, blaZ, fosB-Saur, mph (C), msr(A), sat4, tet(38), mgrA* | 3 (3.6) |
| P23 | *mecA, ermC, fosB-Saur, fusC, tet(38), mgrA* | 1 (1.2) |
| P24 | *mecA, aph(2'')-Ih, blaZ, dfrC, ermC, tet(38), mgrA* | 3 (3.6) |
| P25 | *mecA, blaZ, dfrG, fexA, fosB-Saur, fusC, tet(38), tet(L), mgrA* | 1 (1.2) |
| P26 | *mecA, fosB-Saur, fusC, tet(38), mgrA* | 2 (2.4) |
| P27 | *mecA, blaZ, ermC, tet(38), mgrA* | 1 (1.2) |
| P28 | *ant(4')-Ia, blaZ, bleO, fosB-Saur, fusB, msr(A), tet(38), mgrA* | 2 (2.4) |
| P29 | *mecA, blaZ, dfrG, fosB-Saur, tet(38), mgrA* | 2 (2.4) |
| P30 | *mecA, blaZ, ermC, fusC, tet(38), mgrA* | 3 (3.6) |
| P31 | *mecA, fosB-Saur, lnu(A), tet(38), mgrA* | 1 (1.2) |
| P32 | *mecA, blaZ, dfrG, ermC, fosB-Saur, fusC, tet(38), mgrA* | 3 (3.6) |
| P33 | *mecA, blaZ, fosB-Saur, lnuA, tet(38), mgrA* | 1 (1.2) |
| P34 | *blaZ, fosB-Saur, tet(38), mgrA*  *blaZ, fosB-Saur, tet(38), mgrA* | 1 (1.2) |

Supplementary Table 2: Enterotoxin genes among Staphylococci isolates from meat and patients

| Enterotoxin genes | | No. (%) of Staohylococci isolates | | | | | | |
| --- | --- | --- | --- | --- | --- | --- | --- | --- |
|  |  | Camel (n= 23 ) | Beef (n=14 ) | Lamb (n= 11) | Chicken (n= 13) | Fish (n=15) | Clinical (n= 84) | Total (n= 160) |
| Classic enterotoxins | *sea* | 6 (26) | 1 (7) | 1 (9) | 1 (8) | 0 (0) | 34 (40) | 43 (27) |
|  | *seb* | 4 (17) | 1 (7) | 2 (18) | 2 (15) | 0 (0) | 20 (24) | 29 (18) |
|  | *sed* | 0 (0) | 0 (0) | 0 (0) | 0 (0) | 0 (0) | 3 (4) | 3 (2) |
|  | *see* | 0 (0) | 0 (0) | 0 (0) | 0 (0) | 0 (0) | 1 (1) | 1 (0.5) |
| Other enterotoxins | *sem* | 3 (13) | 3 (21) | 4 (36) | 5 (38) | 9 (60) | 43 (51) | 67 (42) |
|  | *sei* | 4 (17) | 3 (21) | 4 (36) | 4 (31) | 8 (53) | 43 (51) | 66 (41) |
|  | *sen* | 4 (17) | 2 (14) | 4 (36) | 4 (31) | 8 (53) | 40 (48) | 62 (39) |
|  | *seg* | 4 (17) | 2 (14) | 4 (36) | 2 (15) | 8 (53) | 40 (48) | 60 (38) |
|  | *seo* | 4 (17) | 2 (14) | 4 (36) | 2 (15) | 8 (53) | 40 (48) | 60 (38) |
|  | *seu* | 4 (17) | 2 (14) | 4 (36) | 2 (15) | 8 (53) | 40 (48) | 60 (38) |
|  | *sel* | 4 (17) | 1 (7) | 2 (18) | 1 (8) | 0 (0) | 7 (8) | 15 (9) |
|  | *sel*X | 17 (73) | 12 (86) | 8 (72) | 7 (54) | 6 (40) | 79 (94) | 139 (87) |
|  | *ser* | 0 (0) | 1 (7) | 0 (0) | 0 (0) | 0 (0) | 5 (6) | 6 (4) |
|  | *sey* | 1 (4) | 1 (7) | 4 (36) | 1 (8) | 1 (7) | 3 (4) | 11 (7) |
|  | *seh* | 0 (0) | 1 (7) | 0 (0) | 0 (0) | 3 (20) | 2 (2) | 6 (4) |
|  | *sel*27 | 0 (0) | 0 (0) | 0 (0) | 0 (0) | 1 (7) | 2 (2) | 3 (2) |
|  | *sej* | 0 (0) | 0 (0) | 0 (0) | 0 (0) | 0 (0) | 5 (6) | 5 (3) |
|  | *sel*Z | 0 (0) | 0 (0) | 0 (0) | 0 (0) | 0 (0) | 2 (2) | 2 (1) |

Supplementary Table 3: Virulence genes among *S.aureus* isolates from meat and patients

| Gene product | virulence genes | | *S.aureus* Source (Food/clinical) | | | | | | |
| --- | --- | --- | --- | --- | --- | --- | --- | --- | --- |
|  |  |  | Camel (n= 18 ) | Beef (n=12 ) | Lamb (n= 8) | Chicken (n= 7) | Fish (n=8) | Clinical (n= 83) | Total (N= 136) |
| Penicillin-binding protein 2a (PBP2a) | *mec*A | | 6 | – | 1 | 3 | 1 | 80 | 91 |
| Toxic shock syndrome toxin-1 | *tsst-1* | | 2 | – | – | 1 | 3 | 8 | 14 |
|  |  |  |  |  |  |  |  |  |  |
| Panton–Valentine leukocidin | *lukS-PV* | | – | 1 | – | – | – | 23 | 24 |
|  | *lukF-PV* | |  |  |  |  |  |  |  |
| Capsular polysaccharide synthesis enzyme | *cap* | | 18 | 12 | 8 | 7 | 8 | 83 | 136 |
| Alpha-Hemolysin precursor | *hly/hla* | | 18 | 12 | 8 | 7 | 8 | 83 | 136 |
| Fibronectin-binding protein A | *fnbA* | | 3 | – | – | 3 | – | 26 | 32 |
| Cell surface elastin binding protein | *ebp* | | 18 | 12 | 8 | 7 | 8 | 74 | 127 |
| Clumping factor, A fibrinogen-binding protein | *clf* | A | 5 | 7 | 2 | 2 | – | 11 | 27 |
|  |  |  |  |  |  |  |  |  |  |
|  |  |  | 11 | 8 | 2 | 6 | 6 | 59 | 92 |
|  |  | B |  |  |  |  |  |  |  |
| IgG-binding protein | *sbi* | | 18 | 12 | 8 | 7 | 8 | 83 | 136 |
| Glycerol ester hydrolase | *geh* | | 18 | 12 | 8 | 7 | 8 | 83 | 136 |
| Gamma-hemolysin chain II precursor | *hlg* | A | 18 | 12 | 8 | 7 | 8 | 83 | 136 |
|  |  | B | 18 | 12 | 8 | 7 | 8 | 83 | 136 |
|  |  | C | 17 | 12 | 8 | 7 | 7 | 82 | 133 |
| Ser-Asp rich fibrinogen-binding bone sialoprotein-binding protein | *sdr* | E | 12 | 9 | 8 | 6 | 5 | 76 | 116 |
|  |  | D | 12 | 9 | 6 | 6 | 7 | 72 | 112 |
|  |  | C | 13 | 12 | 5 | 7 | 5 | 71 | 113 |
| Type VII secretion system protein | *esa* | A | 18 | 12 | 8 | 7 | 8 | 83 | 136 |
|  |  | B | 18 | 12 | 8 | 7 | 8 | 83 | 136 |
|  |  | C | 13 | 9 | 4 | 6 | 5 | 70 | 107 |
| Type VII secretion system protein | *ess* | A | 18 | 12 | 8 | 7 | 8 | 83 | 136 |
|  |  | B | 18 | 12 | 8 | 7 | 8 | 83 | 136 |
|  |  | C | 13 | 8 | 4 | 6 | 5 | 70 | 106 |
| Type VII secretion system protein | *esx* | A | 18 | 12 | 8 | 7 | 8 | 83 | 136 |
|  |  | B | 13 | 9 | 4 | 6 | 5 | 70 | 107 |
| Intercellular adhesion protein | *ica* | A | 18 | 12 | 8 | 7 | 8 | 83 | 136 |
|  |  | B | 18 | 12 | 8 | 7 | 8 | 83 | 136 |
|  |  | C | 18 | 12 | 8 | 7 | 8 | 83 | 136 |
|  |  | R | 18 | 12 | 8 | 7 | 8 | 83 | 136 |
| Zinc metalloproteinase aureolysin | *aur* | | 17 | 12 | 8 | 7 | 5 | 80 | 129 |
| Triacylglycerol lipase precursor | *lip* | | 18 | 12 | 8 | 7 | 8 | 83 | 136 |
| Delta-hemolysin | *hld* | | 18 | 12 | 8 | 7 | 8 | 83 | 136 |
| Adenosine synthase A | *adsA* | | 18 | 12 | 8 | 7 | 8 | 83 | 136 |
| Serine protease; V8 protease; glutamyl endopeptidase | *ssp* | A | 18 | 11 | 7 | 7 | 8 | 83 | 134 |
|  |  | B | 18 | 12 | 8 | 7 | 8 | 83 | 136 |
|  |  | C | 18 | 12 | 8 | 7 | 8 | 83 | 136 |
| Iron-regulated surface determinant protein | *isd* | A | 18 | 12 | 8 | 7 | 8 | 79 | 132 |
|  |  | B | 18 | 12 | 8 | 7 | 8 | 81 | 134 |
|  |  | C | 18 | 12 | 8 | 7 | 8 | 81 | 134 |
|  |  | D | 18 | 12 | 8 | 7 | 8 | 81 | 134 |
|  |  | E | 18 | 12 | 8 | 7 | 8 | 83 | 136 |
|  |  | F | 18 | 12 | 8 | 7 | 8 | 83 | 136 |
|  |  | G | 18 | 12 | 8 | 7 | 8 | 83 | 136 |
| NPQTN specific sortase B | *srtB* | | 18 | 12 | 8 | 7 | 8 | 83 | 136 |
| Hyaluronate lyase precursor | *hysA* | | 17 | 11 | 5 | 7 | 3 | 75 | 118 |
| Extracellular proteins Map | *map* | | 5 | 7 | 2 | 2 | 2 | 28 | 46 |
| Staphylococcal enterotoxin precursor | *sec* | | 2 | 1 | 2 | 1 | – | 7 | 13 |
|  | *sell* | | 3 | 1 | 2 | 2 | – | 7 | 15 |

Supplementary Table 4: The source of the specimen, the prevalence and percentage of *S. aureus* in clinical samples.

| Clinical specimen | No. of specimen | Percentage |
| --- | --- | --- |
| Urine midstream | 4 | 4.70% |
| Tissue culture | 4 | 4.70% |
| wound swab | 18 | 21.40% |
| Nasal swab | 6 | 7.10% |
| Pus Swab | 9 | 10.70% |
| Groin swab | 2 | 2.30% |
| axilla swab | 4 | 4.70% |
| burn swab | 2 | 2.30% |
| eye swab | 1 | 1.19% |
| Bronchial wash | 3 | 3.50% |
| sputum | 11 | 13% |
| Ascites (peritoneal) Fluid | 1 | 1.19% |
| Tracheal aspirate | 4 | 4.70% |
| Blood culture | 15 | 17.80% |

Supplementary table 5: *S. aureus* clonal complexes with their associated resistance genes, virulence genes, and enterotoxin genes
